# Indications for surgery versus conservative treatment in the management of lumbar disc herniations: A systematic review

**DOI:** 10.1016/j.bas.2025.105619

**Published:** 2025-09-27

**Authors:** Santhosh G. Thavarajasingam, Ahmed Salih, Aksaan Arif, Madhur Varadpande, Pratheeshan Sabeshan, Hariharan Subbiah Ponniah, Sree Kanakala, Srikar R. Namireddy, Daniele S.C. Ramsay, Ahkash Thavarajasingam, Daniel Scurtu, Dragan Jankovic, Andreas Kramer, Florian Ringel

**Affiliations:** a Imperial Brain & Spine Initiative, Imperial College London, London, United Kingdom; b Faculty of Medicine, Imperial College London, London, United Kingdom; c Faculty of Medicine, Medizinische Hochschule Hannover, Hannover, Germany; d Department of Neurosurgery, LMU University Hospital Munich, Munich, Germany; e EANS Spine Section, European Association of Neurosurgical Societies, Belgium

**Keywords:** Lumbar disc herniation, Slipped disc, Surgical indications, Conservative management, Radiculopathy, Motor deficits, Index of qualitative variation, Early surgery, Decision-making criteria, Transition to surgery, Evidence-based guidelines

## Abstract

**Introduction:**

Lumbar disc herniation (LDH) is a leading cause of radiculopathy and low back pain, contributing significantly to global disability. Management strategies include conservative and surgical treatments, but clinical decision-making lacks standardization, particularly in surgical indications, timing, and criteria for transitioning from conservative management.

**Research question:**

What are the surgical indications, criteria for transitioning from conservative to surgical management in LDH, and what role do motor deficits play?

**Material and methods:**

Following PRISMA guidelines, a systematic search across major databases identified 20 studies. Risk of bias was assessed using the Newcastle Ottawa Scale and RoB 1 tools. A qualitative synthesis was conducted, and the Index of Qualitative Variation (IQV) quantified variability in indications.

**Results:**

Among the studies that reported specific indications, imaging-confirmed nerve root compression (reported in 18/20 studies) and severe/refractory pain (reported in 17/20 studies) were the most consistent indications, while thresholds for sensory deficits (reported in 8/20 studies) varied widely. Early surgery (48 h–6 weeks) was associated with superior recovery, particularly for mild/moderate motor deficits graded ≤ MRC 3/4, achieving >90 % recovery rates. Delayed surgery (>6 weeks) resulted in prolonged symptoms and poorer outcomes, especially in severe cases. Transition criteria included a patient-specific combination of failure of conservative therapy (n = 12) after a most frequently 4–6-week trial, neurological progression, and worsening imaging findings. Significant heterogeneity was observed in thresholds for motor and sensory deficits, with high IQV scores for definitions of conservative treatment failure (IQV = 0.96) and motor deficit (IQV = 0.96).

**Discussion and conclusion:**

Significant heterogeneity in surgical indications, timing, and decision-making highlights the urgent need for standardized, evidence-based guidelines to optimize clinical decisions and improve outcomes in LDH management.

## Highlights

• Imaging-confirmed nerve root compression and severe/refractory pain were the most consistent surgical indications.
• Early surgery (48 h–6 weeks) resulted in >90 % recovery rates, particularly for mild/moderate motor deficits (MRC ≤3/4).
• Delayed surgery (>6 weeks) was linked to prolonged symptoms and poorer outcomes, especially in severe cases.
• Transition criteria included failure of 4–6 weeks of conservative therapy, neurological progression, and worsening imaging findings.
• High variability (IQV = 0.98) in thresholds for motor deficits and treatment failure underscores the need for standardized guidelines.

## 1 Introduction

Lumbar disc herniation (LDH) is a prevalent condition, significantly contributing to low back pain and radiculopathy worldwide (Fardon et al., 2014). It is characterized by the displacement of intervertebral disc material, leading to nerve root compression and associated neurological symptoms (Deyo et al., 2006). Management strategies for LDH range from conservative treatments, such as physical therapy and pharmacotherapy, to surgical interventions aimed at decompressing affected neural structures (Chou et al., 2009). The decision to proceed with surgery is complex, often influenced by factors including the severity of neurological deficits, pain intensity, and the patient's response to non-surgical therapies (Weinstein et al., 2008).

In 2022, the North American Spine Society (NASS) published updated clinical guidelines for the diagnosis and treatment of LDH with radiculopathy (Society NAS, 2022). These guidelines recommend surgical intervention for patients exhibiting significant or progressive neurological impairments, intractable pain unresponsive to conservative measures, or specific structural abnormalities identified through imaging (Society NAS, 2022). However, since their publication, several high-quality studies have emerged, such as the work by Thomé et al. (2022), which demonstrated that earlier surgical intervention in LDH patients was associated with superior motor recovery outcomes (Thomé et al., 2022). This evolving body of evidence, including advancements in understanding timing, indications, and patient selection, suggests the need for a re-evaluation of current guidelines to reflect recent findings (Jacobs et al., 2011).

Previous reviews, such as that by Yoon et al. (2021), have addressed similar questions regarding LDH management but often included broader scopes, such as thoracic and cervical herniations, limiting their depth and detail in LDH-specific analyses (Yoon and Koch, 2021). Since LDH is by far the most common and epidemiologically and clinically significant form of herniation, accounting for the majority of surgical and conservative treatment cases, it warrants dedicated focus (Herzog, 1996). By concentrating exclusively on LDH, this review provides a nuanced and targeted assessment of surgical indications, with particular attention to the timing of surgery, the transition from conservative management, and the role of neurological deficits (Jansson et al., 2004). This systematic review aims to inform clinical practice by delineating clear, evidence-based indications for surgery, thereby optimizing patient outcomes and addressing gaps left by existing guidelines and reviews (Gibson and Waddell, 2005).

## 2 Methods

### 2.1 Search strategy and study selection

This systematic review was conducted in accordance with PRISMA guidelines and utilized the SWiM (Synthesis Without Meta-analysis) framework for data synthesis (Moher et al., 2009; Campbell et al., 2020). The search strategy was designed to identify studies reporting on surgical indications for lumbar disc herniation (LDH) and was conducted on August 10, 2024, using four major databases: PubMed, MEDLINE, EMBASE, and Scopus. The strategy was structured into three conceptual blocks. The first block included terms describing lumbar disc herniation, such as “*lumbar disc herniation”*, “*intervertebral disc displacement”*, “*herniated disc”*, and related synonyms. The second block focused on surgical procedures, incorporating terms such as “*surgery”*, “*discectomy”*, “*laminectomy”*, “*microdiscectomy”*, “*spinal fusion”*, and “*surgical decompression”*. The third block addressed surgical indications and clinical presentations, including terms such as “*surgical indication”*, “*timing of surgery”*, “*neurological deficit”*, “*cauda equina”*, “*motor weakness”*, and “*radiculopathy”*. The full search strategy is detailed in Supplementary Table 1. Studies published between 1943 and 2024 were considered, focusing on adult populations undergoing surgical or conservative management for LDH.

Eligibility criteria were based on the PICOS framework (Population, Intervention, Comparison, Outcomes, Study Design) and included studies evaluating surgical indications, timing of surgery, and criteria for transitioning from conservative to surgical management. Exclusion criteria included studies focusing solely on thoracic or cervical herniations, non-English publications, paediatric populations, and non-peer-reviewed articles. Full inclusion and exclusion criteria are detailed in Supplementary Table 2. For trials with multiple reports, only the most recent and complete dataset was included.

Two independent reviewers (SGT and AS) screened titles, abstracts, and full texts. Discrepancies were resolved through consensus or discussion with a third reviewer, AK. Covidence software was used to facilitate the screening process (Innovation).

## 3 Objectives

This review sought to answer four key research questions:

1. What are the indications for surgery in LDH?
2. What is the optimal timing of surgery?
3. What are the criteria for switching from conservative to surgical management?
4. What is the role of motor deficits in determining surgical indications?

### 3.1 Data extraction and quality assessment

Data extraction was conducted manually using a standardized Excel spreadsheet (Supplementary Table 3) and focused on variables such as study characteristics, patient demographics, and surgical indications. Extracted data were systematically organized and presented in the manuscript's main tables and supplementary materials (Supplementary Tables 3 and 4). Risk of bias was assessed independently by two reviewers (AS and AA) using the Newcastle Ottawa Scale (NOS) and RoB 1 (Risk of Bias 1.0) tool with disagreements resolved by SGT (Sterne et al., 2016; Wells BS and O'Connell; Higgins et al., 2011). The level of evidence was evaluated using the Oxford Centre of Evidence-Based Medicine (OCEBM) criteria (Oxford Centre for Evidence-Based Medicine (OCEBM)).

### 3.2 Qualitative synthesis and statistical analysis

The synthesis of evidence followed the SWiM framework, emphasizing narrative synthesis and descriptive statistics. Descriptive statistics summarized key study variables, including study characteristics and extracted outcomes, as presented in the manuscript's main tables (Table 1, Table 2, Table 3) (Campbell et al., 2020). The heterogeneity observed across patient populations, surgical techniques, and follow-up methodologies significantly limited the feasibility of quantitative pooling and formal statistical synthesis.

**Table 1:** Study characteristics of the included studies in this systematic review.

Study	Sample size	Study type & design	Level of Evidence	Country	Type of surgery	Control group	Indication for Surgery	F/U Period	Primary and Secondary Outcomes	Lost To Follow Up	Complications
Gurung et al., 2023	87	Retrospective cohort	2B	UK	Lumbar discectomy	N/A	Symptomatic lumbar disc herniation confirmed by MRI, without progressive neurological deficits or cauda equina syndrome, in patients who had failed conservative treatment	6 months	Primary: Achievement of minimal clinically important difference (MCID) in Oswestry Disability Index (ODI), leg pain Visual Analog Scale (VAS) and back pain VAS. Secondary: Return to work and functional recovery within 6 weeks post-surgery.	N/A	N/A
Motiei-Langroudi et al., 2023	134	Prospective cohort	2B	USA	Unilateral laminotomy and microdiscectomy	Combination of physical therapy, pain medications (NSAIDs, opioids, gabapentin) and epidural steroid injections.	Persistent radicular leg pain despite at least 4–6 weeks of maximal conservative therapy.	N/A	Primary: Validation of a scoring system to predict surgical need; rates of conservative treatment failure; factors predictive of surgery (disc size, location and type)	N/A	N/A
Bailey et al., 2021	128	Randomised controlled trial	1B	Canada	Microdiscectomy	Patient education, pain medications (NSAIDs, acetaminophen, gabapentin), exercised physiotherapy and epidural steroid injections.	Chronic sciatica (duration 4–12 months) due to MRI-confirmed posterolateral disc herniation (L4-L5 or L5-S1) causing radiculopathy and failure of conservative treatment for ≥6 months.	24 months	Primary: VAS for leg pain Secondary: VAS for back pain, ODI, SF-36 physical and mental component scores	Operative group: 25 % (Wells BS and O'Connell) Non-operative: 34 % (Motiei-Langroudi et al., 2023)	Operative group: Superficial wound infection (n = 2), post-op adjacent level condition (n = 1), new-onset postop neuropathic pain (n = 1) Non-operative group (after crossing over to operative): Dural 2Btear (n = 2), super3Bficial wound in3Bfection (n = 1), nerve root injury (n = 2), postop adjacent-level condition (n = 2), new-onset postop neuropathic pain (n = 2)
Thomé et al., 2022	390	Prospective cohort	2B	Austria	Fragmentectomy or microdiscectomy	N/A	Patients with LDH-associated radicular motor deficits, confirmed by MRI or CT imaging, and deemed ineligible for conservative management.	43.4 ± 19.7 months	Primary: Recovery of motor strength measured by the MRC scale	0.3 % (Fardon et al., 2014)	N/A
Gupta et al., 2020	368	Retrospective case control	3B	USA	Microdiscectomy	Pain medications (NSAIDs, gabapentin, pregabalin), epidural steroid injections, and physical therapy	Persistent lumbar radicular pain with MRI-confirmed disc herniation, failing to improve after 6 weeks of conservative management.	48 months	Primary: Whether size of a lumbar disc herniation predicts failure of conservative management and necessitates surgery. Secondary: Interobserver reliability of MRI measurements (size, area)	N/A	N/A
Ostafiński et al., 2020	240	Retrospective case control	3B	Poland	Microdiscectomy	Physiotherapy, lumbar traction, progressive exercises, manual therapy, and injections (epidural steroids, selective transforaminal nerve blocks, and sacroiliac/facet joint injections).	Persistent symptoms despite 6 weeks of conservative treatment. Imaging-confirmed lumbar disc herniation with significant spinal canal stenosis or nerve root compression.	6 months	Primary: Predictors for conservative treatment failure (e.g. percent canal stenosis, presence of leg paraesthesia) Secondary: Development of predictive model to evaluate risk of surgical need.	N/A	N/A
Petr et al., 2019	330	Prospective cohort	2B	Austria	Microdiscectomy	N/A	Acute motor deficits caused by lumbar disc herniation, lasting <48 h or >48 h, with MRI-confirmed diagnosis.	3 months	Primary: Recovery of motor strength measured by the MRC scale, recovery of sensory deficits, and sciatica relief. Secondary: Neurological outcome predictors, including timing of surgery and preoperative motor deficit severity.	N/A	Haemorrhage (n = 2), accidental durotomy (n = 16), infection/delayed wound healing (n = 7), recurrent herniation requiring reoperation (n = 18)
Kim et al., 2016	64	Retrospective case-control	3B	Korea	Microdiscectomy	Patients managed with transforaminal epidural steroid injections (TFESI) combined with medial branch blocks for radicular leg pain and back pain.	Single-level lumbar disc herniation at L4-L5 with persistent radicular leg pain and back pain unresponsive to conservative treatment. Patients had to meet the inclusion criteria, including MRI-confirmed LDH and organized medical records.	12 months	Primary: VAS for leg and back pain, motor deficit recovery using MRC scale; MacNab classification for procedural success Secondary: Radiological factors were assessed as predictors of surgical and non-surgical outcomes.	N/A	None
Motiei-Langroudi et al., 2014	134	Retrospective case control	3B	Iran	Not specified	Physical therapy and medical therapy including NSAIDs, TCAs, long-acting injectable corticosteroids.	Persistent and unresolved pain after completing conservative therapy or intolerable pain despite conservative medical therapy. MRI findings, including thecal sac compression, larger disc fragments, higher Pfirrmann's grades, and specific disc herniation types (extrusion/protrusion), were predictive of surgical need.	N/A	Primary: Factors predicting surgical need, including clinical and MRI parameters such as cerebrospinal fluid (CSF) block, disc fragment size, and herniation location/type.	N/A	N/A
Overdevest et al., 2014	150	Randomised controlled trial	1B	Netherlands	Microdiscectomy	Received information on their condition, advised to continue activities of daily living. If necessary, analgesics were prescribed or guidance from a physical therapist was recommended.	Randomised to received early surgical treatment or conservative therapy. Study inclusion criteria was patients aged 18–65 years old presenting with sciatica with motor neurological deficit due to LDH persisting 6–12 weeks. LDH was confirmed with MRI and symptom severity justified surgical treatment.	1 year (repeat standardized neurological examinations at baseline, 8, 26 and 52 weeks)	Primary: Motor deficit through manual muscle testing and graded according to the MRC scale. Secondary: Roland disability score, VAS leg pain, Satisfactory overall recovery, LDH, cross-sectional area of the spinal canal.	Two patients were lost to follow-up early (conservative only).	N/A
Choi et al., 2013	46	Retrospective case-control	3B	Korea	Microdiscectomy	Transforaminal epidural steroid injections, selective nerve root blocks, and active physical therapy.	Severe motor weakness caused by lumbar disc herniation, with MRI-confirmed nerve compression correlating with clinical symptoms.	12 months (evaluations at 1,3,6 and 12)	Primary: Recovery of motor weakness using MRC scale Secondary: VAS for back and leg pain, and ODI for functional disability.	N/A	N/A
Haugen et al., 2012	466	Prospective cohort	2B	Norway	N/A - limited details	Information about sciatica and disc herniation, back exercises, physical therapy and pain medication. Information and treatment were given on an individual basis to the patients at each centre.	The decision about surgery was made for individual patients at each centre and no standardized criteria were established for surgical treatment.	2 years (questionnaires sent after 3, 6, 12 and 24 months)	Primary: Maine-Seattle Back Questionnaire (MSBQ). Non-success was defined as a MSBQ score of more than or equal to 5. Secondary: Sciatica Bothersomeness Index (SBI). Non-success was defined as a SBI score of more than or equal to 7.	88 (18.89 %) at 2-year follow-up	N/A
Lurie et al., 2013	307	Retrospective cohort	2B	USA	Open discectomy with decompression of the affected nerve root	Physical therapy, education and counseling with home exercise instruction and non-steroidal anti-inflammatory drugs.	Radicular pain for at least six weeks with a positive nerve root tension sign and/or a neurologic deficit, and a confirmatory cross-sectional imaging study demonstrating a herniated disc at the level and side corresponding to their symptoms.	4 years	Primary: AAOS/Modems modified version of the Oswestry Disability Index (ODI). Secondary: SF-36 Bodily Pain (BP) and Physical Functioning (PF) scales, Sciatica Bothersomeness Scale, Back Pain Bothersomeness scale.	N/A	N/A
Thomas et al., 2007	497	Prospective Cohort	2B	Canada	Microdiscectomy	N/A	Severe leg pain and/or weakness with a specific dermatomal/myotomal pattern, with congruent findings from disc protrusion on CT or MRI.	Minimum of 1 year	*Primary*: NASS NSS (NASS Neurogenic Symptoms Score) *Secondary*: PDS (NASS Pain and Disability Score), SF-36	126 (25.4 %) at 15 month follow up	N/A
Carlisle et al., 2005	88	Retrospective Case Control	3B	USA	Microdiscectomy	Non-surgical management included physical therapy, NSAIDs, oral steroids, spinal injections, 2Bacupuncture, and2B chiropractic1B care for longer2B than 3 months	Intractable leg or back pain, significant functional impairments, or unsatisfactory improvement after 3 months of conservative care.	N/A	Primary: % Canal compromise + disc herniation size	N/A	N/A
Rothoerl et al., 2002	219	Prospective	2B	Germany	Conventional discectomy	N/A	Surgery on monosegmental lumbar disc herniations for the first time	9.9 months	Primary: Prolo Scale	7 % lost to follow-up	N/A
Kavuncu et al., 2001	85	Retrospective	2B	Turkey	Limited details	N/A	Low back pain and/or sciatica, positive straight leg raising test (SLRT), motor or sensory loss with or without loss of tendon reflexes and diagnosis by myelography, computerized tomography or magnetic resonance 93. imaging.	29.8 months (average)	Primary: VAS scale	105 (58.3 %) lost to follow -up	N/A
Weinstein et al., 2006	501	Randomised controlled trial	1B	USA	Open discectomy	“Usual care”: active physical therapy, education/counseling with home exercise instruction, NSAIDs, physicians were encouraged to individualise treatment to the patient	Radicular pain and evidence of nerve-root irritation with a positive nerve-root tension sign (straight leg or a corresponding neurologic deficit	2 years	Primary: SF-36 Bodily Pain and physical function; ODI (American Academy of Orthopaedic Surgeons MODEMS version of the Oswestry Disability Inde) Secondary: patient- self-reported improvement, work status, satisfaction with current symptoms and care, and sciatica severity as measured by the Sciatica Bothersomeness Index.	29 (5.8 %) lost to follow-up	Intraoperative: dural tear/spinal fluid leak (n = 10), vascular injury (n = 1). Postoperative: wound infection (n = 2)
Weinstein et al., 2006	743	Prospective	2B	USA	Open discectomy	“Usual care”: active physical therapy, education/counseling with home exercise instruction, NSAIDs, physicians were encouraged to individualise treatment to the patient	Symptoms and confirmatory signs of lumbar radiculopathy that persisted for at least 6 weeks, who had disc herniation at a corresponding level and side on imaging	2 years	Primary: SF-36 Bodily Pain and physical function; ODI (American Academy of Orthopaedic Surgeons MODEMS version of the Oswestry Disability Index) Secondary: patient- self-reported improvement, work status, satisfaction with current symptoms and care, and sciatica severity as measured by the Sciatica Bothersomeness Index.	24 (3.2 %) lost to follow-up	Dural tear (n = 9) reoperation (n = 32) by 1 year and (n = 42) of cases at 2 years.
Peul et al., 2007	283	Randomised controlled trial	1B	Netherlands	Microdiscectomy	“Usual care’: reassurance/education, encourage activity; analgesics per guidelines; physiotherapy if fear-avoidance; offer surgery if symptoms persisted ≥6 months or sooner for intractable pain/progressive deficit.	Severe sciatica 6–12 weeks with MRI-confirmed symptomatic disk herniation	1 year (assessed at 2, 4, 8, 12, 26, 38, 52 weeks).	**Primary:** RDQ disability, VAS leg pain, and time to patient-reported recovery (Likert). **Secondary:** SF-36; Sciatica Frequency & Bothersomeness Index; health-perception VAS; neurological exam; functional/economic observations.	Overall: 7/283 (2.5 %) Early surgery: 4/141 (2.8 %) Conservative: 3/142 (2.1 %)	Operative group: dural tear (n = 2), wound haematoma (n = 1). Conservative group: N/A

In Table 1, the study characteristics of the included studies in this systematic review (n = 20) are summarized. The following variables were extracted: study (author and date of publication), sample size, study type and design (randomised controlled trial, prospective cohort, retrospective study), OCEBM level of evidence, country of origin, follow-up period, surgical intervention (microdiscectomy or open discectomy), control group treatments (e.g., physical therapy, NSAIDs, epidural steroid injections), indications for surgery, primary and secondary outcomes, loss to follow-up (LTFU), and complications. **ODI**: Oswestry Disability Index; **VAS**: Visual Analog Scale; **SF-36**: Short Form-36 Health Survey; **NSAIDs**: Non-Steroidal Anti-Inflammatory Drugs; **RCT**: Randomised Controlled Trial; **MRI**: Magnetic Resonance Imaging.

**Table 2:** Detailed summary of the surgical indications from each of the included studies in this systematic review.

Study	Main Indication	Motor deficit	Other neurological deficit	Severe/Refractory Pain	Imaging	Failure of conservative therapy	Miscellaneous indications	Main conclusion	Risk of bias
Gurung et al., 2023	Persistent lumbar radicular pain unresponsive to conservative treatment	No	No	Yes - Measured using VAS scores for leg pain (>7.5 mean baseline).	Yes - MRI-confirmed LDH	N/A	N/A	Surgery provided significant improvements in leg pain, back pain, and functional outcomes (ODI, VAS). Time to surgery beyond 6 months did not significantly worsen outcomes, suggesting that the timing of intervention could remain flexible in the absence of worsening neurological deficits.	NOS D1: Low D2: High D3: Low Overall: Some concern
Motiei-Langroudi et al., 2023	Persistent radicular leg pain unresponsive to conservative therapy.	No	No	Yes – severity not explicitly quantified.	Yes – MRI-confirmed single level lumbar disc herniation	Yes - Lack of improvement after 4–6 weeks of conservative treatment, including physical therapy, NSAIDs, gabapentin, opioids, and/or epidural steroid injections.	N/A	A scoring system based on MRI findings (disc/fragment size, location, and type) can predict surgical need. Patients with higher scores (≥4) had >90 % likelihood of requiring surgery, helping to avoid unnecessarily prolonged conservative treatment.	NOS D1: Low D2: Some concern D3: Low Overall: Low
Bailey et al., 2021	Chronic sciatica with radicular symptoms significantly affecting quality of life.	Yes – Asymmetrical motor weakness affecting the lower limb.	Yes - Sensory deficits (numbness and tingling) and reflex changes (asymmetrical decrease)	No	Yes - MRI evidence of posterolateral disc herniation at L4-L5 or L5-S1 compressing the corresponding nerve root.	Yes - Persistent symptoms after 6 months of standardized nonoperative care, including physiotherapy, oral analgesics, and up to three epidural steroid injections.	N/A	Microdiscectomy resulted in greater improvements in leg pain and physical function compared to nonoperative care at 6 months and 1 year. However, these advantages diminished at 2 years due to crossover effects, highlighting the potential for early surgery in selected cases.	ROB-1 D1: Low D2: High D3: High D4: Unclear D5: Low D6: Unclear
Thomé et al., 2022	Radicular motor deficits with severity ranging from mild to severe (MRC ≤2/5)	Yes – Classified with MRC scale.	No.	No.	Yes - Either MRI or CT confirmed LDH.	N/A	N/A	Early surgical intervention (within 3 days for severe deficits and 8 days for mild deficits) leads to better recovery of motor function. Timing of surgery was critical, with delays beyond these windows significantly reducing the likelihood of full recovery.	NOS D1: Low D2: Some concerns D3: Low Overall: Low
Gupta et al., 2020	Persistent lumbar radicular pain unresponsive to conservative management for at least 6 weeks.	No.	No.	Yes - Persistent and severe radicular pain, though not quantified using specific pain scales.	Yes – MRI findings included: Herniation size as a percentage of spinal canal area and Disc herniation location.	Yes – Lack of symptom improvement after 6 weeks of conservative management, including combinations of: physical therapy, NSAIDs, gabapentin, pregabalin or steroid injections.	N/A	The size of the herniated disc does not predict the likelihood of failing conservative management. The findings highlight the importance of patient-specific factors rather than disc size in determining surgical need.	NOS D1: Low D2: High D3: Low Overall: Some concern
Ostafiński et al., 2020	Persistent lumbar radicular pain and/or sensory deficits unresponsive to conservative management.	No.	Yes – Sensory deficits, including leg paraesthesia.	Yes - Persistent leg pain resistant to conservative measures.	Yes - MRI parameters included Type of herniation and Percent canal stenosis ratio.	Yes – Lack of significant symptom improvement after 6 weeks of conservative management, including combinations of physical therapy, exercise regimens and pain management using steroid injections and nerve blocks.	N/A	Surgical intervention was most strongly predicted by a percent canal stenosis ratio ≥23 % and the presence of leg paraesthesia. These findings suggest that combining clinical and imaging factors can optimize decision-making for lumbar disc herniation management.	NOS D1: Low D2: High D3: Some concerns Overall: Some concerns
Petr et al., 2019	Moderate to severe motor deficits.	Yes - Patients had moderate (MRC Grade 3) or severe (MRC Grades 0–2) motor deficits.	No.	No.	Yes – MRI and/or CT scans confirmed. Parameters measured include Location of herniation and Confirmation of nerve compression and herniation severity.	N/A	N/A	Immediate surgery (within 48 h) for acute moderate to severe motor deficit resulted in significantly faster recovery compared to delayed surgery. Recovery of motor and sensory deficits was strongly associated with early surgical intervention.	NOS D1: Low D2: High D3: Low Overall: Some concerns
Kim et al., 2016	Persistent, intractable radicular pain and/or motor/sensory deficits after conservative treatment.	Yes – evaluated using the MRC scale but no specific grading for intervention was mentioned.	Yes - however no specific grading for intervention mentioned.	Yes - severe radicular pain was assessed using VAS, but no specific grading for intervention was mentioned.	Yes – MRI-confirmed LDH at L4-L5. Disc herniation length and area was measured.	N/A	N/A	Patients with disc herniation length >6.31 mm and refractory radicular pain achieved better outcomes with surgery compared to nerve block therapy.	NOS D1: Low D2: High D3: Low Overall: Low
Motiei-Langroudi et al., 2014	History of lower back pain or related radicular leg pain.	No.	No.	Yes -surgery was performed for patients with intolerable pain that did not improve with conservative management.	Yes – MRI confirmed diagnosis. Parameters measured include: Pfirrmann grade and type of disc herniation.	Yes – if symptoms persisted despite minimum of 4 weeks of conservative therapy, including physical and medical management (NSAIDs, tricyclic antidepressants, and corticosteroids),	In those with intolerable pain despite conservative therapy, the decision was made sooner to undergo surgery.	Conservative management is recommended initially for all patients with LDH. However, larger herniation fragments, higher Pfirrmann grades, and the presence of a CSF block predict conservative treatment failure and the need for surgery. Prolonged conservative management beyond 4–8 weeks may worsen outcomes.	NOS D1: Some concerns D2: High D3: Some concerns Overall: High
Overdevest et al., 2014	Sciatica accompanied by motor deficits.	Yes – MRC grade 3 or 4.	No.	Yes – sciatica due to LDH persisting 6–12 weeks.	Yes – LDH confirmed on MRI	Yes - disabling sciatica persisting for 6 months after the patient was randomised for conservative treatment.	N/A	Early surgery resulted in a faster recovery of motor deficit accompanying sciatica compared with prolonged conservative treatment, but the difference was no longer significant at the final follow-up examination at 1 year.	ROB-1 D1: Unclear D2: High D3: High D4: Low D5: Low D6: High
Choi et al., 2013	Motor deficit	Yes – no specific indicator for surgery was mentioned.	No.	No.	Yes – MRI confirmation of LDH.	N/A	N/A	Surgical treatment for motor weakness caused by LDH lead to a rapid recovery in the short-term, especially 1 month. Early and proper surgical treatment for motor weakness provides rapid alleviation.	NOS D1: Low D2: High D3: Low Overall: Some concern
Haugen et al., 2012	Individually determined by an orthopaedic surgeon in each centre with no standardized criteria.	Yes - abnormal if there was reduced extension or flexion of the knee, ankle, or big toe; abnormal tiptoe or heel walking; or a positive Trendelenburg test.	N/A	N/A	Yes - severe pain was part of the clinical profile. Pain intensity in the leg VAS,	Yes - back exercises, physical therapy, and pain medication was part of the conservative treatment protocol. The duration of conservative therapy prior to surgery was not explicitly stated but implied to be several weeks.	N/A	Surgery resulted in slightly better outcomes than conservative care at 1 year but showed diminishing advantages at 2 years. Muscular weakness and abnormal reflexes were predictors of poorer outcomes, emphasizing the need for broader patient assessments beyond physical symptoms.	NOS D1: Low D2: Some concerns D3: Low Overall: Low
Lurie et al., 2013	Radicular pain for at least six weeks with a positive nerve root tension sign and/or a neurologic deficit.	Yes - specific motor grading was not detailed	Yes - classification was based on imaging findings (e.g., nerve root compression/displacement). No explicit scales or metrics used.	Yes – radicular pain for at least six weeks with a positive nerve root tension sign.	Yes – MRI-confirmed LDH. Parameters recorded include extent of thecal sac and nerve root compression, and disc morphology.	N/A	N/A	Patients with ≥1/3 thecal sac compression or nerve root compression/displacement derive greater surgical benefit compared to those with minimal compression or Modic type 1 changes. Imaging predictors were critical in guiding surgical decisions for LDH.	NOS D1: Low D2: High D3: Low Overall: Low
Thomas et al., 2007	Leg pain and or weakness.	No.	No.	Yes – pain focused on radicular symptoms, with many patients presenting with severe pain unresponsive to treatment	Yes – confirmatory MRI and CTs were used to confirm disc protrusion.	N/A	N/A	Surgical and non-surgical groups demonstrated similar improvements in health-related quality of life, though surgical outcomes showed statistically significant but clinically minor benefits for secondary measures (NASS PDS and SF-36 mental health domains). Persistent disability was noted in both groups at follow-up.	NOS D1: Low D2: Some concerns D3: Low Overall: Low
Carlisle et al., 2005	Intractable leg or back pain, significant functional impairments, or unsatisfactory improvement after 3 months of conservative care.	Yes – all patients had a level of myotomal weakness, although it is not clear how this was qualified	No.	Yes - pain was described as intractable back or leg pain unresponsive to conservative measures over 3 months	Yes – MRI findings included herniated disc area, canal cross-sectional area and % canal compromise	Yes – defined as failure of physical therapy, NSAIDs, oral steroids, spinal injections and acupuncture after 3 months	N/A	Surgery was more likely in patients with larger disc herniation areas and higher percent canal compromise, as shown on MRI, when conservative management failed. Central herniations particularly showed a higher correlation with surgical necessity.	NOS D1: Low D2: High D3: Some concerns Overall: Some concerns
Rothoerl et al., 2002	Patients who underwent surgery on monosegmental lumbar disc herniations for the first time.	N/A	N/A	Yes – pain levels were measured using a VAS, with the mean duration of pain recorded as 86 days	N/A	N/A	N/A	Delays beyond 60 days led to statistically worse outcomes in terms of symptom relief and recovery, underscoring the importance of timely intervention.	NOS D1: Low D2: Some concerns D3: Low Overall: Low
Kavuncu et al., 2001	Low back pain and/or sciatica, positive straight leg raising test (SLRT), motor or sensory loss with or without loss of tendon reflexes and diagnosis by myelography, computerized tomography or magnetic resonance imaging.	Yes - motor with or without loss of tendon reflexes and diagnosis by myelography	Yes - sensory loss was considered	Yes – lower back pain and siatica were indications	Yes - CT, or MRI was used to confirm the location and nature of the herniation corresponding to clinical symptoms.	N/A	Positive straight leg raising test	Surgery led to significant improvements in motor strength, sensory deficits, and lumbar mobility compared to conservative treatment. However, surgically treated patients reported higher pain and disability scores at follow-up, emphasizing the need for individualized treatment plans based on patient-specific clinical factors.	NOS D1: Low D2: High D3: Some concerns Overall: Some concerns
Weinstein et al., 2006	Radicular pain and evidence of nerve-root irritation with a positive nerve-root tension sign or a corresponding neurologic deficit	Yes – the trial had a generalised inclusion criteria rather than a specific one for surgical candidates. The generalised inclusion criteria included neurological deficit which encompasses motor weakness	Yes – the trial had a generalised inclusion criteria rather than a specific one for surgical candidates. The generalised inclusion criteria included neurological deficit which encompasses motor weakness	Yes - pain was classified as radicular pain radiating below the knee for lower lumbar herniations or into the anterior thigh for upper lumbar herniations.	Yes, MRI and CT were used to confirm disc herniation (classifying into protrusion, extrusion, or sequestered fragment as well as by herniation level)	Yes - Patients had to demonstrate persistent symptoms for at least 6 weeks despite these efforts. Conservative treatments were individualized and included physical therapy (67 %), epidural injections (42 %), and medications (NSAIDs, oral steroids, and narcotics).	N/A	Both surgical and nonoperative groups improved over two years. While surgical intervention provided faster relief, particularly for leg pain, the long-term differences were modest. Patients benefited most when treatment selection aligned with clinical and imaging findings, emphasizing the necessity for individualized decision-making.	ROB-1 D1: Low D2: High D3: High D4: Unclear D5: Low D6: High
Weinstein et al., 2006	All men and women who had symptoms and confirmatory signs of lumbar radiculopathy that persisted for at least 6 weeks, who had disk herniation at a corresponding level and side on imaging, who were considered surgical candidates.	Yes – the trial had a generalised inclusion criteria rather than a specific one for surgical candidates. The generalised inclusion criteria included neurological deficit which encompasses motor weakness	Yes – the trial had a generalised inclusion criteria rather than a specific one for surgical candidates. The generalised inclusion criteria included neurological deficit which encompasses motor weakness	Yes - pain was classified as radicular pain radiating below the knee for lower lumbar herniations or into the anterior thigh for upper lumbar herniations.	Yes - MRI and CT were used to confirm disc herniation (classifying into protrusion, extrusion, or sequestered fragment as well as by herniation level)	Yes - Conservative care included physical therapy (73 %), epidural injections (50 %), NSAIDs (58 %), and opioid analgesics (49 %). Patients had to fail these treatments for at least 6 weeks.	N/A	Both surgical and nonoperative treatments led to significant improvements in outcomes over two years. Surgical patients experienced more rapid and greater improvements in pain, function, and disability scores, as reflected in measures like the SF-36 and Oswestry Disability Index. The study highlights that surgical intervention is effective for appropriately selected patients but emphasizes shared decision-making due to similar long-term outcomes.	NOS D1: Low D2: Some concerns D3: Low Overall: Low
Peul et al., 2007	Severe sciatica persisting 6–12 weeks	N/A	N/A	Yes - patients had disabling radicular pain lasting 6–12 weeks	Yes - MRI-confirmed lumbar disc herniation	Yes - only included if symptoms persisted ≥6 weeks despite conservative care	N/A	Early surgery resulted in faster pain relief and recovery, but at 1 year outcomes were similar between early surgery and prolonged conservative care.	ROB-1 D1: Low D2: High D3: Low D4: Low D5: Low D6: High

In Table 2, surgical indications reported across the included studies (n = 20) are summarized. The following categories of indications were extracted and analysed: motor deficits, refractory pain, imaging findings, failure of conservative therapy, other miscellaneous criteria, main conclusions of the study, as well as risk of bias scoring as per Newcastle Ottawa Scale (NOS) or Risk of Bias 1 tool (RoB-1), the latter for randomised control trials. **MRC**: Medical Research Council (muscle strength grading scale); **VAS**: Visual Analog Scale; **LDH**: Lumbar Disc Herniation; **MRI**: Magnetic Resonance Imaging; per Newcastle Ottawa Scale, **NOS**; Risk of Bias 1, **RoB-1.**

**Table 3:** Detailed summary of the results from each of the included studies in this systematic review on the timing of surgery and switching from conservative to surgery.

Study	Conservative Treatment Attempted	Conservative Treatment Duration	Timing of Surgery	Criteria for Early Surgery	Criteria for Delayed Surgery	Transition Criteria Surgery to Conservative	Definition of Failed Conservative Therapy	Time to Surgery After Conservative Therapy Failure	Influence of Motor Deficits	Main Conclusion	Risk of Bias
Gurung et al., 2023	Yes	22.5 weeks	Both early (<6 months) and delayed (≥6 months) were analysed.	<6 months	≥6 months	N/A	N/A	N/A	Patients with progressive motor deficits, foot drop, or cauda equina syndrome were excluded from the study, so motor deficits were not a direct influence on timing.	Early surgery (<6 months) resulted in a higher proportion of patients achieving clinically significant improvement in leg pain VAS (90.2 % vs. 80.8 %) and ODI (60.7 % vs. 42 %) compared to delayed surgery. However, statistical significance was not achieved, suggesting that delayed surgery may not adversely impact outcomes if no progressive neurological deficits are present.	D1: Low D2: High D3: Low Overall: Some concern
Motiei-Langroudi et al., 2023	Yes	Minimum 4–6 weeks	Delayed - surgery was performed after conservative treatment failure in patients with persistent radicular leg pain.	N/A	Conservative treatment failure	Lack of symptom improvement after completing the full conservative treatment protocol.	Lack of symptom improvement after 4–6 weeks of physical therapy, pain medications, and/or epidural steroid injections.	Mean time 24 ± 7.1 days	Patients with profound or progressive motor deficits were excluded from the study. Motor deficits did not influence surgical decision-making.	A scoring system based on MRI findings (disc/fragment size, location, and type) effectively predicted the need for surgery. The findings support avoiding unnecessarily prolonged conservative management beyond 4–8 weeks for patients likely to require surgical intervention.	D1: Low D2: Some concern D3: Low Overall: Low
Bailey et al., 2021	Yes	Minimum of 6 months before considering surgery in the nonoperative group.	Delayed - Surgery was performed after failure of conservative management or based on crossover from nonoperative care to surgical treatment.	N/A	Persistent radicular leg pain and/or functional impairment after at least 6 months of conservative care.	Lack of improvement in radicular leg pain, functional disability, and/or quality of life after completing the full nonoperative care protocol.	Persistence of sciatica despite 6 months of standardized nonoperative treatment, including physiotherapy and medication.	Patients crossed over to surgery after a mean of 6–12 months, depending on individual circumstances.	Motor deficits were present in some patients; however, they were not the primary indication for surgery or a major focus in the study.	Microdiscectomy was superior to standardized nonoperative care for chronic sciatica caused by lumbar disc herniation at 6 months and 1 year but showed no statistically significant differences in outcomes at 2 years. A significant proportion of patients crossed over to surgery, emphasizing the limitations of prolonged conservative management.	ROB-1 D1: Low D2: High D3: High D4: Unclear D5: Low D6: Unclear
Thomé et al., 2022	No	N/A	Both early (≤3 days) and delayed (>3 days) were analysed.	≤3 days	>3 days	N/A	N/A	N/A	Motor deficits were the primary determinant of surgical timing. Severe deficits required urgent intervention, while mild deficits could tolerate short delays without compromising outcomes.	Early surgical intervention (≤3 days) significantly improves motor recovery for severe and moderate deficits. For mild motor deficits, surgery within 8 days ensures better outcomes. Timing is critical, with delayed intervention associated with poorer recovery rates.	D1: Low D2: Some concerns D3: Low Overall: Low
Gupta et al., 2020	Yes	Minimum 6 weeks	Delayed – After failure of conservative therapy.	N/A	Persistent lumbar radicular pain and/or functional impairment despite 6 weeks of conservative treatment.	Lack of significant symptom improvement following the full course of conservative management.	Persistent symptoms despite ≥6 weeks of nonoperative treatment.	N/A	Patients with profound or progressive motor neurological deficits were excluded from the study, so motor deficits were not an influencing factor.	The size of the herniated disc as a percentage of the spinal canal does not predict failure of conservative treatment. Surgical decision-making should prioritize patient-specific symptoms and responses rather than imaging metrics.	D1: Low D2: High D3: Low Overall: Some concern
Ostafiński et al., 2020	Yes	6 weeks	Delayed - Surgery was performed after conservative treatment failure, focusing on cases with persistent symptoms or poor quality of life.	N.A	Conservative treatment failure	Lack of significant symptom improvement after 6 weeks of conservative management.	Lack of significant improvement after a 6-week conservative management protocol.	N/A	Patients with significant motor or sphincter deficits were excluded from the study. Motor deficits did not influence the decision-making process in this study.	Leg paraesthesia and a spinal canal stenosis ratio of ≥23 % were independent predictors of conservative treatment failure. These findings support surgical intervention for patients meeting these criteria after 6 weeks of conservative management.	D1: Low D2: High D3: Some concerns Overall: Some concerns
Petr et al., 2019	No	N/A	Early (<48 h) versus delayed (>48 h)	Motor deficits lasting ≤48 h	Motor deficits persisting >48 h.	N/A	N/A	N/A	Significant; earlier surgery (<48 h) resulted in faster and more complete recovery of moderate to severe motor deficits (MRC 0–3), with statistically significant differences in outcomes at discharge, 6 weeks, and 3 months.	Immediate surgery within 48 h of motor deficits onset leads to substantially faster recovery for moderate to severe motor deficits. Mild motor deficits showed no significant difference in outcomes regardless of timing. The study suggests urgent surgery for acute motor deficits is both effective and safe.	D1: Low D2: High D3: Low Overall: Some concerns
Overdevest et al., 2014	Yes	12 months	Early – 1.8 weeks after randomisation and cancelled only in case of spontaneous improvement of symptoms (in 7 (10 %) of the 70 assigned to early surgery)	Randomised to either receive early surgery or prolonged conservative treatment.	Intolerable pain during conservative treatment.	If disabling sciatica persisted for 6 months after the patient was offered conservative treatment, surgery was offered.	Persistent radicular leg pain and back pain despite at least 6 months of conservative management	N/A	In this current analysis, motor deficits ranged from moderate (MRC grade 4) to severe (MRC grade 3).	Early surgery resulted in a faster recovery of motor deficit accompanying sciatica compared with prolonged conservative treatment, but the difference was no longer significant at the final follow-up examination at 1 year.	ROB-1 D1: Unclear D2: High D3: High D4: Low D5: Low D6: High
Carlisle et al., 2005	Yes	3 months	Delayed – Surgery was performed after conservative treatment failure, and were excluded from the study if they had no supervised conservative treatment or prior spinal surgery	N/A	Conservative treatment failure	Lack of significant symptom improvement after 3 months of onset	Persistent symptoms despite at least 4 weeks of conservative treatment or intolerable pain during this period.	N/A	Whilst all patients included had a degrees of myotomal weakness, the effect on conservative treatment failure is unclear	Surgery was more likely in patients with larger disc herniation areas and higher percent canal compromise, as shown on MRI, when conservative management failed. Central herniations particularly showed a higher correlation with surgical necessity.	D1: Low D2: High D3: Some concerns Overall: Some concerns
Rothoerl et al., 2002	Yes	Up to 2 months	Early (<60 days) vs Delayed (>60 days)	Early surgery was not explicitly defined as a treatment strategy in the study but was recommended based on outcome analyses.	Patients with symptoms persisting beyond 60 days (e.g., sensory or motor deficits, severe pain)	N/A	Disabling sciatica persisting for 6 months after the patient was randomised for conservative treatment.	N/A	Motor deficits were present in 54 % of patients but did not significantly influence the timing of surgery or outcomes in this study.	Patients operated on within 2 months of symptom onset had better outcomes compared to those undergoing delayed surgery (>60 days). Conservative treatment should be limited to 2 months, and surgery should be considered for non-responders.	D1: Low D2: Some concerns D3: Low Overall: Low
Weinstein et al., 2006	Yes	6 weeks	Delayed – Surgery was performed after conservative treatment failure	N/A	Conservative treatment failure	Lack of significant symptom improvement after 6 weeks of onset	N/A	N/A	Whilst there was a significant proportion of patient with motor weakness reported at baseline, the effect of this on switch from conservative to surgery is unclear	Both surgical and nonoperative groups improved over two years. While surgical intervention provided faster relief, particularly for leg pain, the long-term differences were modest. Patients benefited most when treatment selection aligned with clinical and imaging findings, emphasizing the necessity for individualized decision-making.	ROB-1 D1: Low D2: High D3: High D4: Unclear D5: Low D6: High
Weinstein et al., 2006	Yes	6 weeks	Delayed – Surgery was performed after conservative treatment failure	N/A	Conservative treatment failure	Lack of significant symptom improvement after 6 weeks of onset	N/A	N/A	Whilst there was a significant proportion of patient with motor weakness reported at baseline, the effect of this on switch from conservative to surgery is unclear	Both surgical and nonoperative treatments led to significant improvements in outcomes over two years. Surgical patients experienced more rapid and greater improvements in pain, function, and disability scores, as reflected in measures like the SF-36 and Oswestry Disability Index. The study highlights that surgical intervention is effective for appropriately selected patients but emphasizes shared decision-making due to similar long-term outcomes.	D1: Low D2: Some concerns D3: Low Overall: Low
Peul et al., 2007	Yes	6–12 weeks before randomisation; conservative strategy then continued up to 6 months	Early (within 2 weeks) vs Delayed (offered after 6 months if needed)	Randomised to early surgery; microdiscectomy scheduled within 2 weeks	Persisting sciatica at 6 months after randomisation; earlier if intractable pain or progressive neurologic deficit	N/A	Persistence of sciatica to 6 months after randomisation, or earlier deterioration (increasing leg pain not responsive to medication or progressive neurologic deficits)	Mean 18.7 weeks to surgery among those in the conservative arm who later had surgery	Patients with paralysis or strength < against gravity were excluded; progressive neurologic deficit triggered earlier surgery	Early surgery provided faster relief and recovery; 1-year outcomes were similar between strategies	ROB-1 D1: Low D2: High D3: Low D4: Low D5: Low D6: High Overall: Low

**In**Table 3, **the findings related to the timing of surgery and the criteria for transitioning from conservative to surgical treatment across the included studies (n = 20) are summarized. The following variables were extracted: author and year, timing of surgery, definition of early surgery, definition of delayed surgery, criteria for transitioning from conservative to surgical treatment, time to surgery after conservative treatment failure, and key findings. VAS**: Visual Analog Scale; **NRS**: Numeric Rating Scale; **ODI**: Oswestry Disability Index; **MRI**: Magnetic Resonance Imaging; **IQV**: Index of Qualitative Variation.

Instead, a qualitative approach was adopted. Studies were grouped thematically based on their primary surgical indication, such as motor deficits, failure of conservative management, or timing of surgery. Findings were synthesized using a standardized metric: the frequency (n) of studies reporting each indication or threshold.

The Index of Qualitative Variation (IQV) was then applied to these frequencies to measure the diversity of surgical indications across studies. The IQV quantifies the dispersion of categorical variables by calculating the proportion of responses in each category relative to the total number of studies that reported data for that variable. Values for the IQV range from 0 to 1, where 0 indicates no diversity (all responses fall into a single category) and 1 represents maximum diversity (responses are evenly distributed across all categories). In this review, IQV was applied to assess the variability in reported surgical indications, with values exceeding 0.7 denoting high variability. This analysis provided a structured approach to understanding the consistency or variability in surgical indications, offering insights into the diversity of clinical practices.

All analyses and visualizations were performed using R (version 4.4.1) in accordance with the SWiM framework (R: A language and environment). The R scripts used for analysis are available on request via GitHub.

## 4 Results

**Fig. 1:**
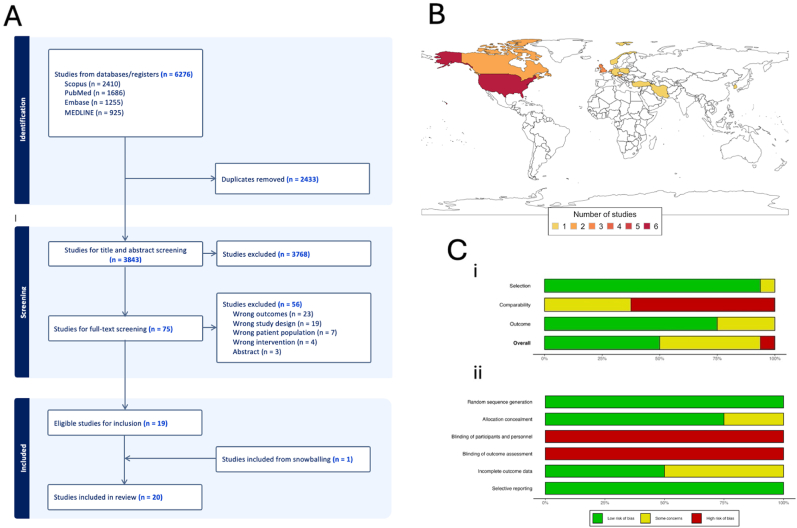
In Fig. 1A, the preferred Reporting Items for Systematic Reviews and Meta-Analyses (PRISMA) flowchart outlining the study selection process is shown. In Fig. 1B a world map indicated the origin of publications included in this study (n = 20) (Thomé et al., 2022; Gurung et al., 2023; Motiei-Langroudi et al., 2014, 2023; Bailey et al., 2021; Gupta et al., 2020; Ostafiński et al., 2020; Petr et al., 2019; Kim et al., 2016; Overdevest et al., 2014; Choi et al., 2013; Haugen et al., 2012; Lurie et al., 2013; Thomas et al., 2007; Carlisle et al., 2005; Rothoerl et al., 2002; Kavuncu et al., 2001; Weinstein et al., 2006a, 2006b; Peul et al., 2007). The countries are coloured according to whether n = 1, 2, 3, 4, 5 or 6 studies from these countries have been included in this systematic review. The legend at the bottom indicates the color coding. Following countries are coloured: Austria (n = 2), Canada (n = 1), Germany (n = 1), Iran (n = 1), South Korea (n = 2), Netherlands (n = 2), Norway (n = 1), Poland (n = 1), Turkey (n = 1), UK (n = 1), United States of America (n = 6). Figure Ci shows the risk-of-bias summary for randomised studies (n = 4), presented as a bar chart displaying the distribution of risk-of-bias judgments across the domains of the Risk of Bias 1 (RoB-1) tool, expressed as percentages. The overall risk of bias, representing the combined judgments across all domains, is displayed at the bottom. Figure Cii shows the corresponding summary for non-randomised studies (n = 16), using the Newcastle-Ottawa Scale, with domain-specific distributions and an overall risk-of-bias assessment presented similarly.

Of 6276 records identified, 2433 duplicates were removed. Subsequently 3843 records were screened by title and abstract, of which 3768 were excluded. The remaining 75 full-text articles were assessed for eligibility, and 56 were excluded with reasons (Fig. 1A), resulting in 19 studies being eligible for inclusion (Thomé et al., 2022; Gurung et al., 2023; Motiei-Langroudi et al., 2014, 2023; Bailey et al., 2021; Gupta et al., 2020; Ostafiński et al., 2020; Petr et al., 2019; Kim et al., 2016; Overdevest et al., 2014; Choi et al., 2013; Haugen et al., 2012; Lurie et al., 2013; Thomas et al., 2007; Carlisle et al., 2005; Rothoerl et al., 2002; Kavuncu et al., 2001; Weinstein et al., 2006a, 2006b). One additional study was identified following ‘snowballing’ of references (Peul et al., 2007). Therefore, 20 studies were included in this review. Study characteristics, including design, country, sample size, and follow-up duration, are summarized in Table 1. The geographic distribution of study origins is presented in Fig. 1B, indicating that most studies originated from North America and Europe, with the United States contributing the largest number (n = 6). The risk of bias assessments across all studies, evaluated using the NOS and ROB1 tools, are summarized in Fig. 1C, showing variability in methodological rigor across domains. Surgical indications and criteria, including motor deficits, refractory pain, imaging findings, and failure of conservative therapy, are detailed in Table 2. Key findings on the timing of surgery and transitions from conservative to surgical treatment are synthesized in Table 3.

**Fig. 2:**
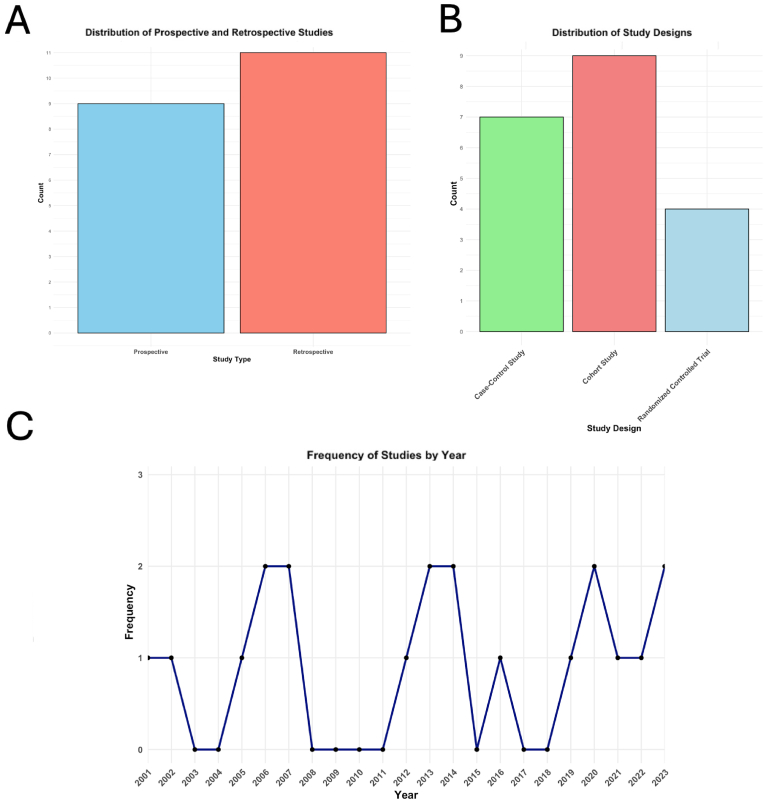
In Fig. 2A a bar plot visualizes the number of prospective (n = 9) and retrospective (n = 11) studies included in the systematic review (n = 20) (Thomé et al., 2022; Gurung et al., 2023; Motiei-Langroudi et al., 2014, 2023; Bailey et al., 2021; Gupta et al., 2020; Ostafiński et al., 2020; Petr et al., 2019; Kim et al., 2016; Overdevest et al., 2014; Choi et al., 2013; Haugen et al., 2012; Lurie et al., 2013; Thomas et al., 2007; Carlisle et al., 2005; Rothoerl et al., 2002; Kavuncu et al., 2001; Weinstein et al., 2006a, 2006b; Peul et al., 2007). In Fig. 2B a bar plot visualizes the number of included studies (n = 20) that are cohort studies (n = 9), case-control studies (n = 7) and randomised controlled trials (n = 4). In Fig. 2C–a line plot displays the number of studies for the following years of publication: 2001 (n = 1), 2002 (n = 1), 2005 (n = 1), 2006 (n = 2), 2007 (n = 2), 2012 (n = 1), 2013 (n = 2), 2014 (n = 2), 2016 (n = 1), 2019 (n = 1), 2020 (n = 2), 2021 (n = 1), 2022 (n = 1), and 2023 (n = 2). Each year is indicated as black circle, and the circles are connected by a line to visualise the trend more clearly.

The included studies comprised 4 randomised controlled trials (RCTs), 9 cohort studies, 7 case-control studies, with follow-up durations ranging from 6 weeks to over 4 years (Fig. 2A and B). Sample sizes varied significantly, from as few as 10 patients to 743 patients, resulting in a combined sample size of 3689 patients (Fig. 2A). The frequency of studies published per year is depicted in Fig. 2C, showing increased publication activity in the last decade, reflecting growing research interest in LDH management.

Out of the 20 included studies, the risk of bias was assessed using two tools: the modified Newcastle Ottawa Scale (NOS) and the RoB 1 (Risk of Bias 1.0), the latter being for randomised trials only. For studies assessed with NOS, 16 studies were categorised, of which 7 were rated as having ‘some concerns’ of bias, and 1 study rated as ‘high risk’. The remaining 4 studies were evaluated using the RoB-1 tool, with each randomised trial having one or more domains classified as ‘high risk’, particularly regarding performance and detection bias. Further domain-specific and overall assessments are summarized in Supplementary Figs. 1 and 2. Evidence levels were assessed using the OCEBM criteria, with studies classified as level 1b (n = 4), 2b (n = 9), and 3b (n = 7). Further information is provided in Supplementary Table 4.

### 4.1 Indications for surgery

**Fig. 3:**
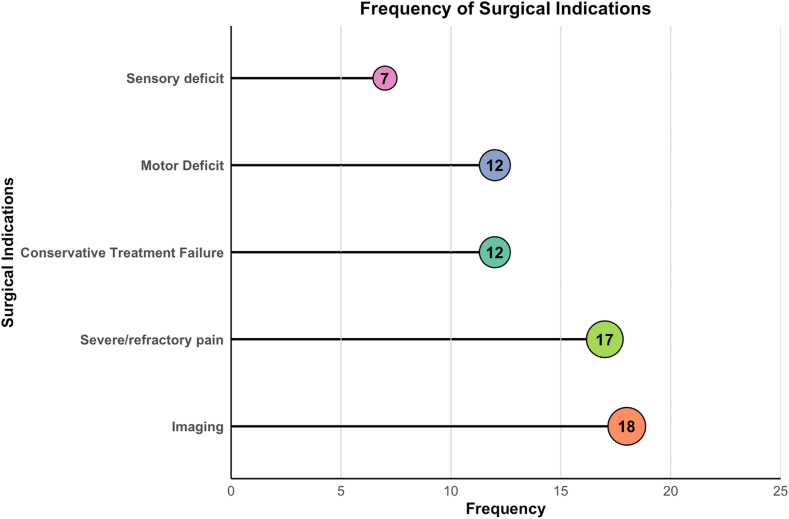
In Fig. 3, a lollipop plot illustrating the frequency of surgical indications for LDH across the included studies (Thomé et al., 2022; Gurung et al., 2023; Motiei-Langroudi et al., 2014, 2023; Bailey et al., 2021; Gupta et al., 2020; Ostafiński et al., 2020; Petr et al., 2019; Kim et al., 2016; Overdevest et al., 2014; Choi et al., 2013; Haugen et al., 2012; Lurie et al., 2013; Thomas et al., 2007; Carlisle et al., 2005; Rothoerl et al., 2002; Kavuncu et al., 2001; Weinstein et al., 2006a, 2006b; Peul et al., 2007). The x-axis represents the frequency of studies, while the y-axis lists the surgical indications: “Sensory Deficit” (n = 7), “Motor Deficit” (n = 12), “Conservative Treatment Failure” (n = 12), “Severe/Refractory Pain” (n = 17), and “Imaging” (n = 18). Coloured circles mark the frequencies at the end of each line, emphasizing that “Imaging” and “Severe/Refractory Pain” are the most frequently reported indications, followed by “Conservative Treatment Failure."

Surgical indications for LDH were stratified into three primary categories: neurological deficit, intractable pain, and imaging findings, as shown in Table 2. Across studies, the decision to proceed with surgery was influenced by a combination of these factors, with emphasis varying depending on the study design and patient population. Fig. 3 provides an overview of the frequency of these surgical indications across the included studies. Imaging findings (n = 18) and severe or refractory pain (n = 17) were the most frequently reported indications. This high frequency was matched by high consistency, as quantified by a low Index of Qualitative Variation (IQV = 0.36), indicating most studies used similar radiological criteria. Other commonly reported factors included failure of conservative treatment (n = 12), motor deficits (n = 12), and sensory deficits (n = 7).

### 4.2 Neurological deficit

Neurological deficit, particularly motor weakness, emerged as the most consistent indication for surgery. Kim et al. reported that progressive motor weakness was a primary driver for surgical intervention, with imaging confirming nerve root compression in 87 % of cases (Kim et al., 2016). Studies such as Overdevest et al. emphasized that the presence of a new or worsening neurological deficit was prioritized over other factors, with motor strength ≤ MRC grade 3 being a threshold for immediate surgery in 92 % of patients (Overdevest et al., 2014). Conversely, Petr et al. noted variability in clinical practice, with some centers opting for a more conservative approach to mild deficits, highlighting the heterogeneity in surgical decision-making (Petr et al., 2019).

#### 4.2.1 Intractable pain

Unrelenting radicular pain was the second most common indication for surgery. Bailey et al. identified pain refractory to at least six weeks of conservative management as a major determinant, supported by visual analog scale (VAS) scores >7 in surgical candidates (Bailey et al., 2021). Similarly, Motiei-Langroudi et al. found that patients reporting persistent, disabling pain despite pharmacological and physical therapy interventions were 70 % more likely to transition to surgery compared to those with manageable pain levels (Motiei-Langroudi et al., 2023). However, Haugen et al. argued that pain alone, without neurological compromise, often led to variable outcomes, particularly in the absence of definitive imaging findings (Haugen et al., 2012). Weinstein et al. demonstrated that surgery provided superior long-term outcomes for pain relief and functional recovery compared to conservative care, with significant differences observed in the Spine Patient Outcomes Research Trial (SPORT) cohort (Weinstein et al., 2006a, 2006b).

### 4.3 Imaging findings

Advanced imaging modalities, particularly MRI, played a pivotal role in guiding surgical decision-making. Thomé et al. highlighted that patients with herniation types such as extrusion or sequestration were more likely to undergo surgery due to the higher likelihood of nerve root compression (Thomé et al., 2022). Similarly, Motiei-Langroudi et al. reported that higher Pfirrmann grades and herniation fragment sizes correlated strongly with surgical intervention, with a predictive accuracy of 85 % for conservative treatment failure (Motiei-Langroudi et al., 2023).

In summary, imaging-confirmed nerve root compression (n = 18) and severe or refractory pain (n = 17) emerged as the most consistent and predominant indications for surgical intervention in LDH patients, as illustrated in Table 2 and Fig. 3. These primary factors were frequently combined to guide clinical decision-making across the studies, underscoring their critical role in the management of LDH. However, significant variability was observed in the thresholds for motor and sensory deficits, with some studies prioritizing immediate surgery for moderate deficits while others adopted a more conservative approach.

#### 4.3.1 Timing of surgery

Optimal timing for surgical intervention in lumbar disc herniation (LDH) is a critical determinant of patient outcomes. Across the included studies, definitions of early and delayed surgery varied, with timing assessed in relation to the onset of symptoms and the progression of neurological deficits. Table 3 summarizes the timing criteria and associated outcomes.

#### 4.3.2 Early surgery

Early surgical intervention, commonly defined as occurring within 48 h to six weeks of symptom onset, was consistently associated with superior outcomes in patients presenting with moderate to severe motor deficits or unrelenting pain. Petr et al. demonstrated that patients treated within 48 h achieved the highest recovery rates, particularly in motor function, with significant reductions in neurological deficit severity (p < 0.01) (Petr et al., 2019). Similarly, Thomé et al. reported a 20 % improvement in functional recovery for patients undergoing early surgery compared to delayed intervention, underscoring the importance of prompt surgical care in mitigating chronic pain and disability risks (p = 0.03) (Thomé et al., 2022).

In patients with acute motor deficits, particularly those graded ≤ MRC 2, early intervention was particularly critical. Evidence indicated that recovery rates exceeded 90 % when surgery was performed within 48–72 h (Petr et al., 2019; Overdevest et al., 2014). For moderate deficits (MRC 3), early surgery demonstrated faster recovery times and improved quality of life, although long-term outcomes occasionally converged with delayed surgery (Gupta et al., 2020; Overdevest et al., 2014).

#### 4.3.3 Delayed surgery

Delayed surgery, typically defined as beyond six weeks of symptom onset or conservative management, showed more variable results. While Overdevest et al. observed similar long-term functional outcomes between early and delayed surgery groups, the delay was associated with extended periods of pain and reduced quality of life during conservative treatment (p = 0.01) (Overdevest et al., 2014). Conversely, Gupta et al. found that delayed surgery after failed conservative therapy led to poorer recovery rates, particularly in patients with severe motor or sensory deficits (Gupta et al., 2020). These findings highlight the potential risks of deferring surgical intervention in cases of progressive symptoms or significant baseline impairments. Across randomised evidence, Peul et al. scheduled early microdiscectomy within ∼2 weeks and showed that early surgery halves the time to recovery versus a strategy of prolonged conservative care with optional surgery (Peul et al., 2007). Interestingly, 1-year disability and pain outcomes converged between strategies, demonstrating that many patients ultimately recover well without immediate intervention (Peul et al., 2007).

In summary, these findings suggest that surgical timing should be specific to presentation severity and patient preference. Early surgical intervention remains essential for patients with severe neurological symptoms and progressive deficits, whereas those with tolerable pain and no progression can safely trial conservative care for 4–6 weeks. In this latter group, surgery primarily accelerates recovery and return to function rather than altering long-term outcomes. As ever, shared decision-making is crucial to balance short-term symptom relief, occupational needs, and patient values.

### 4.4 Role of motor deficits

**Fig. 4:**
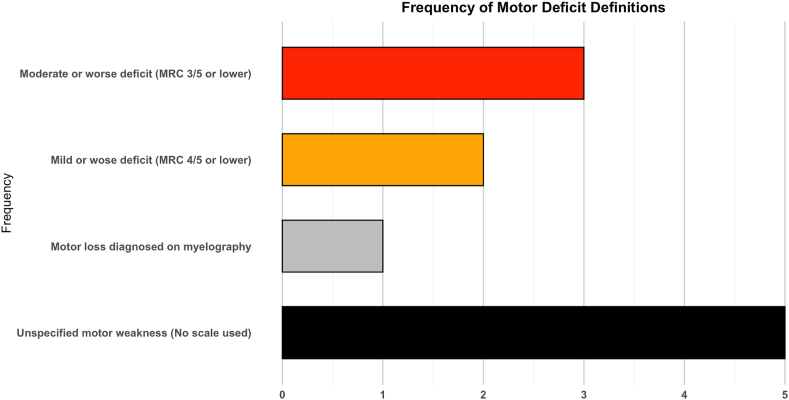
In Fig. 4, a horizontal bar plot showing the frequency of various motor deficit definitions used in the included studies (Thomé et al., 2022; Gurung et al., 2023; Motiei-Langroudi et al., 2014, 2023; Bailey et al., 2021; Gupta et al., 2020; Ostafiński et al., 2020; Petr et al., 2019; Kim et al., 2016; Overdevest et al., 2014; Choi et al., 2013; Haugen et al., 2012; Lurie et al., 2013; Thomas et al., 2007; Carlisle et al., 2005; Rothoerl et al., 2002; Kavuncu et al., 2001; Weinstein et al., 2006a, 2006b; Peul et al., 2007). The x-axis indicates the number of studies, and the y-axis categorizes the types of motor deficits defined. The definitions are “Moderate or Worse Deficit (MRC 3/5 or Lower)" (n = 3), “Mild or Worse Deficit (MRC 4/5 or Lower)" (n = 2), “Motor Loss Diagnosed on Myelography” (n = 1), and “Unspecified Motor Weakness (No Scale Used)" (n = 5). Bars are color-coded as follows: orange for “Moderate or Worse Deficit,” red for “Mild or Worse Deficit,” black for “Motor Loss Diagnosed on Myelography,” and grey for “Unspecified Motor Weakness."

Motor deficits were a key consideration in determining the need for surgery in LDH Across the included studies, findings highlighted the importance of assessing the severity and progression of deficits using standardized grading systems, particularly the Medical Research Council (MRC) scale. Table 2, Table 3 provide a detailed breakdown of findings stratified by motor deficit severity and timing of intervention. Motor deficits were frequently assessed using the Medical Research Council (MRC) grading scale, but Fig. 4 underscores the variability in how deficits were defined across studies. While moderate deficits (MRC 3/5) and mild deficits (MRC 4/5) were equally represented, definitions of motor loss or asymmetrical weakness varied, with some studies relying on clinical diagnosis without standardized grading.

#### 4.4.1 Severe deficits (MRC Grade ≤2)

Patients with severe motor deficits, characterized by significant paresis or complete loss of function, demonstrated the greatest benefit from early surgical intervention. Kögl et al. reported that surgery within 48–72 h led to motor recovery rates exceeding 90 %, with patients regaining substantial strength and mobility (p < 0.01) (Petr et al., 2019; Overdevest et al., 2014). Similarly, Thomé et al. found that delayed surgery for severe deficits resulted in lower recovery rates and a higher risk of persistent neurological impairment, emphasizing the urgency of immediate intervention in these cases (p = 0.002) (Thomé et al., 2022).

#### 4.4.2 Moderate deficits (MRC Grade 3)

For patients with moderate deficits, evidence suggested that early surgery provided faster recovery and improved short-term outcomes. Overdevest et al. reported a significant advantage in functional improvement at 12 weeks when surgery was performed promptly, though long-term outcomes at 12 months converged with those of delayed intervention groups (p = 0.03) (Overdevest et al., 2014). However, Gupta et al. noted variability in recovery rates depending on patient-specific factors, such as the duration of symptoms and underlying comorbidities, which may influence the decision to delay or proceed with surgery (Gupta et al., 2020).

#### 4.4.3 Mild deficits (MRC Grade 4–5)

Patients with mild motor deficits, defined as slight weakness without significant functional impairment, were predominantly managed conservatively. Most studies, including Choi et al. supported an initial conservative approach, with surgery reserved for cases demonstrating symptom progression or failure to improve after a trial of nonoperative management (p = 0.04) (Choi et al., 2013). Motiei-Langroudi et al. highlighted that the majority of patients with mild deficits responded well to conservative care, with only 18 % transitioning to surgery within six weeks (Motiei-Langroudi et al., 2023).

#### 4.4.4 Progressive motor deficits

Several studies emphasized the critical importance of monitoring for progression in motor deficits, regardless of initial severity. Thomé et al. found that patients with worsening motor deficits had significantly better outcomes when transitioned to surgery within two weeks of symptom progression (p < 0.001) (Thomé et al., 2022). Similarly, Petr et al. reported that delayed intervention in cases of progressive deficits was associated with a higher likelihood of incomplete recovery and residual weakness (p = 0.02) (Petr et al., 2019).

The severity and progression of motor deficits are critical factors in deciding surgical intervention for LDH. Patients with severe deficits (MRC Grade ≤2) benefit most from early surgery, achieving recovery rates exceeding 90 % when operated on within 48–72 h, which minimizes the risk of irreversible damage. For moderate deficits (MRC Grade 3), early surgery leads to faster short-term recovery, although long-term outcomes may be similar to delayed surgery. Mild deficits (MRC Grades 4–5) are typically managed conservatively unless symptoms worsen. Consistent use of standardized grading systems like the MRC scale is essential for timely and accurate diagnosis. These findings highlight that early identification and prompt surgery for severe or progressing motor deficits are vital for optimizing patient outcomes, underscoring the importance of considering both severity and progression in clinical decision-making.

### 4.5 Transitioning from conservative to surgical treatment

**Fig. 5:**
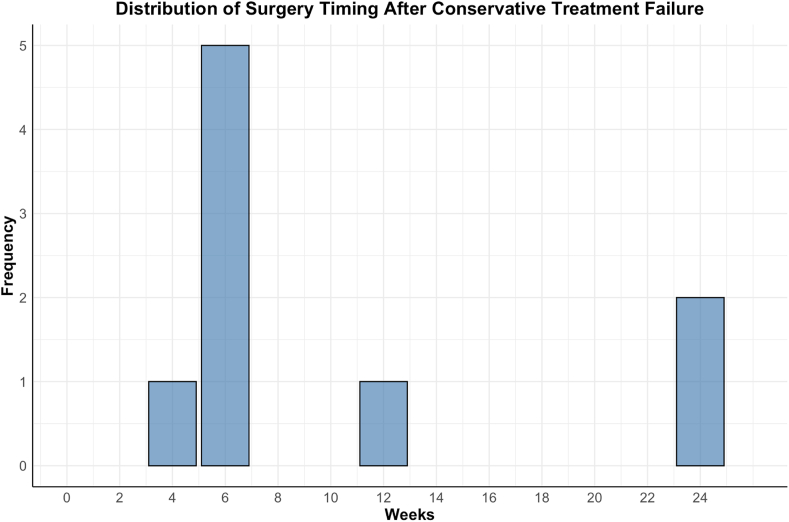
In Fig. 5, a vertical bar plot visualizes the distribution of surgery timing after conservative treatment failure across all included studies (Thomé et al., 2022; Gurung et al., 2023; Motiei-Langroudi et al., 2014, 2023; Bailey et al., 2021; Gupta et al., 2020; Ostafiński et al., 2020; Petr et al., 2019; Kim et al., 2016; Overdevest et al., 2014; Choi et al., 2013; Haugen et al., 2012; Lurie et al., 2013; Thomas et al., 2007; Carlisle et al., 2005; Rothoerl et al., 2002; Kavuncu et al., 2001; Weinstein et al., 2006a, 2006b; Peul et al., 2007). The x-axis represents the weeks after conservative treatment failure, while the y-axis displays the frequency of studies reporting surgery at each time point. The majority of studies (n = 5) reported surgery being performed around the 6-week mark, with smaller frequencies noted at 4 weeks (n = 1), 12 weeks (n = 1), and 24 weeks (n = 1).

The decision to transition from conservative to surgical treatment for LDH involves a multifaceted evaluation, integrating clinical, radiological, and patient-reported factors. This section synthesizes findings from 15 studies outlined in Table 3, focusing on key thresholds and criteria for surgical transition. Fig. 5 illustrates the distribution of surgery timing after conservative treatment failure across included studies, with the majority of interventions performed around the 6-week mark (n = 5). Fewer studies reported interventions at 4 weeks (n = 1), 12 weeks (n = 1), and 24 weeks (n = 1), highlighting variability in clinical practice.

#### 4.5.1 Failure of conservative management

Failure of conservative therapy, defined by a lack of symptomatic improvement, was the most widely reported criterion for transitioning to surgery. Motiei-Langroudi et al. found that 80.6 % of patients improved with conservative management, while non-responders transitioned to surgery within an average of 24 ± 7.1 days (p < 0.05) (Motiei-Langroudi et al., 2023). Similarly, the SPORT trial reported that 70 % of patients with unresolved symptoms opted for surgery within three months, achieving significant symptom relief postoperatively (Weinstein et al., 2006a, 2006b). Gupta et al. supported a 4–6-week threshold, noting that earlier transitions correlated with superior postoperative outcomes in 68 % of non-responders (Gupta et al., 2020). In Peul et al. the conservative strategy formally offered surgery at 6 months unless intractable pain or progressive neurologic deficit triggered earlier surgery, yet 39 % still crossed over at a median of 18.7 weeks (Peul et al., 2007). It is important to note a large symptom burden prompts earlier transition to surgery.

#### 4.5.2 Progression of neurological deficits

Neurological deterioration during conservative management was a critical indication for surgical intervention. Thomé et al. observed that patients with progressive motor weakness transitioned to surgery within two weeks of symptom worsening achieved better outcomes compared to those with delayed transitions (p < 0.001) (Thomé et al., 2022). Overdevest et al. emphasized that postponing surgery despite progressing deficits increased the risk of incomplete recovery and residual deficits (p = 0.02) (Overdevest et al., 2014).

#### 4.5.3 Imaging-confirmed worsening

Radiological evidence of worsening pathology, such as increased herniation size or nerve root compression, frequently prompted surgical transition. Petr et al. reported that 75 % of patients exhibiting worsening MRI findings underwent surgery, correlating positively with symptom progression (p < 0.05) (Petr et al., 2019). Motiei-Langroudi et al. highlighted that high Pfirrmann grades and fragment sizes were predictive of conservative management failure, with a predictive accuracy of 85 % (Motiei-Langroudi et al., 2023).

#### 4.5.4 Persistent pain and disability

Intractable radicular pain unresponsive to conservative measures was another common trigger for surgical intervention. In Bailey's RCT of chronic sciatica, early microdiscectomy produced greater improvements in pain and function at 6 and 12 months compared with standardized nonoperative care, achieving a mean VAS reduction of 2.5 ± 0.6 points (p < 0.001) and 2.1 ± 0.7 points (p < 0.001) respectively (Bailey et al., 2020). At 2 years, these benefits persisted but were diminished and no longer exceeded the minimal clinically important difference, likely due to 38 % crossover from the nonoperative group (Bailey et al., 2021). Haugen et al. similarly noted that patients with sustained disability despite adherence to conservative protocols benefited significantly from surgery, with an odds ratio of 2.97 (95 % CI 1.75–5.04) for improved functional recovery (Haugen et al., 2012).

#### 4.5.5 Timing and transition thresholds

Most studies identified a 4–6-week timeframe as the critical window for conservative treatment failure. However, earlier intervention was often recommended for patients with severe symptoms or rapid neurological deterioration. Kim et al. reported that patients transitioning to surgery within six weeks of conservative failure experienced significantly better outcomes than those undergoing delayed surgery (p = 0.03) (Kim et al., 2016).

In summary, the evidence suggests that the transition from conservative to surgical treatment for LDH should be individualized based on clinical symptoms, radiological findings, and patient responsiveness to conservative therapy. While a 4–6-week trial of conservative management is commonly used as a threshold, earlier surgical intervention is warranted for patients experiencing severe pain, progressive neurological deficits, or worsening imaging findings. This approach ensures timely surgical care for those unlikely to benefit from continued conservative treatment, optimizing patient outcomes and minimizing the risk of prolonged disability.

#### 4.5.6 Variability in practise

**Fig. 6:**
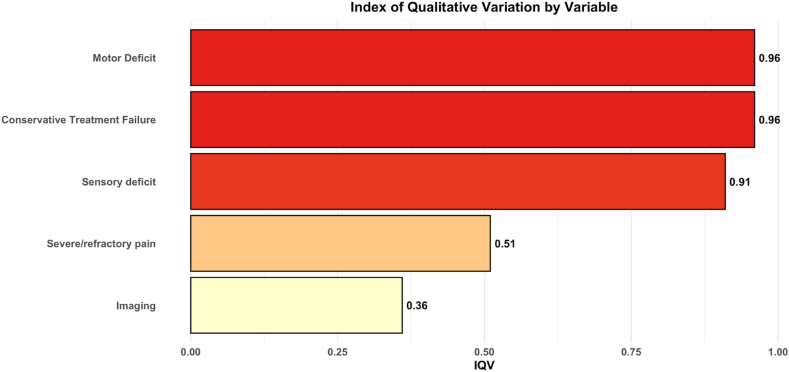
In Fig. 6, a horizontal bar chart depicting the Index of Qualitative Variation (IQV) for different surgical indications for LDH (Thomé et al., 2022; Gurung et al., 2023; Motiei-Langroudi et al., 2014, 2023; Bailey et al., 2021; Gupta et al., 2020; Ostafiński et al., 2020; Petr et al., 2019; Kim et al., 2016; Overdevest et al., 2014; Choi et al., 2013; Haugen et al., 2012; Lurie et al., 2013; Thomas et al., 2007; Carlisle et al., 2005; Rothoerl et al., 2002; Kavuncu et al., 2001; Weinstein et al., 2006a, 2006b; Peul et al., 2007). The chart includes the following variables: “Motor Deficit” (IQV = 0.96), “Conservative Treatment Failure” (IQV = 0.96), “Sensory Deficit” (IQV = 0.91), “Severe/Refractory Pain” (IQV = 0.51) and “Imaging” (IQV = 0.36).

The findings of this review reveal striking variability in surgical indications, timing of surgery, and clinical decision-making for LDH. The Index of Qualitative Variation (IQV), a measure ranging from 0 (no variability) to 1 (maximum variability), quantifies the heterogeneity observed across surgical indications (Fig. 6). Motor deficits (IQV = 0.96) and conservative treatment failure (IQV = 0.96) exhibited the highest levels of variability, followed by sensory deficits (IQV = 0.91). In contrast, severe or refractory pain (IQV = 0.51) and imaging findings (IQV = 0.36) demonstrated greater consistency. This variability is further reflected in Table 2, Table 3, which demonstrate inconsistency in defining surgical thresholds, timing criteria, and decision-making frameworks. The high degree of clinical heterogeneity quantified by the IQV is compounded by methodological heterogeneity, as shown by the variable risk of bias across studies (Supplementary Figs. 1 and 2). This limits the strength of any pooled conclusions and underscores that the findings represent a synthesis of diverse and often low-to-moderate quality evidence. Collectively, these findings highlight the pervasive heterogeneity in clinical decision-making and study design, which poses challenges to synthesizing robust evidence for standardized clinical guidelines.

## 5 Discussion

Synthesizing data from 20 studies and over 3600 patients, this review collates the most recent evidence on surgical indications, timing, and the transition from conservative to surgical management for lumbar disc herniation. Our findings reveal that imaging-confirmed nerve root compression and severe or refractory pain are the most consistent and predominant indications for surgical intervention. Early surgery, particularly for patients with severe motor deficits (MRC ≤2), is consistently associated with better outcomes, including faster recovery and a reduced risk of permanent neurological damage. However, substantial variability exists in the thresholds for motor and sensory deficits, the timing of surgical intervention, and the criteria for transitioning from conservative therapy.

Our review identifies imaging-confirmed nerve root compression and severe or refractory pain as the most consistent indications for surgical intervention in LDH patients, aligning with the North American Spine Society (NASS) guidelines (Society NAS, 2022). However, thresholds for motor and sensory deficits vary significantly across studies, reflecting a lack of standardization in practice. Kim et al. (2022) observed variability in institutional interpretations of radiological findings, which often influenced decisions for surgical intervention (Kim et al., 2016). Similarly, Yoon et al. (2021) highlighted substantial inter-practitioner differences in defining “severe” pain or motor deficits as indications for surgery (Yoon and Koch, 2021). These findings underscore the need for clearer, evidence-based definitions to guide clinical decision-making. Emerging patient-centered frameworks, such as those discussed by Gurung et al. (2023), advocate for integrating patient-reported outcome measures (PROMs) with clinical and radiological findings to refine surgical indications (Gurung et al., 2023). This approach may help standardize decision-making while addressing patient-specific concerns and preferences.

Early surgical intervention, typically defined as surgery within six weeks of symptom onset, was consistently associated with superior neurological recovery. Our findings reinforce the conclusions of Thomé et al. (2022), which demonstrated that early surgery significantly enhances motor recovery and reduces the likelihood of chronic pain (Thomé et al., 2022), and Peul et al. (2007), who showed faster pain relief and earlier return to function with early surgery, though one-year outcomes converged (Peul et al., 2007). Importantly, while early intervention can expedite recovery, many patients with acute radiculopathy will gradually improve with conservative management alone. Thus, in the absence of red-flag symptoms, early intervention may not always allow sufficient time for nonoperative treatments to take effect. In our included studies, a four-to-six-week trial of conservative care was the most common threshold before surgery was considered in patients without red-flag features. Decisions around timing should therefore be guided by symptom severity, occupational demands, and patient preference rather than an expectation of superior long-term outcomes.

The lack of a universally accepted definition for “early” versus “delayed” surgery persists as a critical challenge, with studies such as Gupta et al. (2021) attributing delays to institutional disparities in access and referral patterns (Gupta et al., 2020). Addressing this will require harmonized prospective trials that stratify patients by symptom duration and severity, enabling the establishment of timing thresholds tailored to clinical outcomes and patient preferences. Interestingly, a novel framework proposed by Gurung et al. (2023) advocates for “surgical readiness scores,” combining symptom progression, imaging, and patient comorbidities to optimize the timing of surgery (Gurung et al., 2023). Such tools may bridge the gap between theoretical recommendations and practical clinical decision-making, particularly in resource-limited settings.

Motor deficits remain a debated surgical indication, with variability in definitions and outcomes complicating consensus. While severe deficits (MRC ≤2) were uniformly identified as requiring prompt intervention, moderate deficits (MRC 3) demonstrated mixed outcomes depending on surgical timing. Studies by Overdevest et al. (2021) and Petr et al. (2019) underscore the importance of individualized decision-making in these cases (Petr et al., 2019; Overdevest et al., 2014). However, inconsistent use of grading systems, such as the MRC scale, limits comparability across studies and highlights the need for standardized scoring to guide clinical protocols. Incorporating novel imaging modalities, such as quantitative MRI, could enhance the objectivity of motor deficit assessment. For example, recent advances in nerve root mapping and diffusion tensor imaging have shown promise in correlating radiological findings with functional outcomes, providing a more granular understanding of neurological recovery potential (Motiei-Langroudi et al., 2014). These technologies may complement traditional grading systems, offering a more nuanced approach to surgical planning.

The criteria for transitioning from conservative to surgical management remain inconsistent, reflecting broader gaps in the literature. Our findings echo those of Motiei-Langroudi et al. (2020), who identified imaging-confirmed worsening and failure of conservative therapy as key determinants for surgery (Motiei-Langroudi et al., 2014). However, studies, such as Ostafinski et al. emphasize the integration of patient-reported metrics, such as the Oswestry Disability Index (ODI), to better capture the multidimensional nature of disease progression (Ostafiński et al., 2020). Future research should prioritize the development of predictive models combining patient demographics, patient-reported metrics, symptom trajectories, and imaging biomarkers to refine decision-making frameworks. Additionally, efforts should focus on creating uniform definitions for conservative treatment failure to reduce the observed heterogeneity in practice.

The variability observed in this review, quantified using the Index of Qualitative Variation (IQV), is consistent with prior reviews highlighting heterogeneity in LDH management. For example, Yoon et al. (2021) reported similar inconsistencies in surgical indications, attributing them to differences in study methodologies, patient demographics, and institutional practices (Yoon and Koch, 2021). However, this review extends these findings by systematically quantifying variability across key clinical domains, providing a structured framework for understanding the diversity in practice. Emerging technologies, such as machine learning algorithms, offer opportunities to standardize practice by identifying patterns in clinical decision-making. A recent study by Motiei-Langroudi et al. (2023) demonstrated that predictive models trained on large datasets could accurately stratify patients based on surgical need, potentially reducing variability in management approaches (Motiei-Langroudi et al., 2023).

While this review provides a comprehensive synthesis of the evidence, several limitations must be acknowledged. First, the included studies were predominantly observational, with a moderate to serious risk of bias, limiting the robustness of pooled conclusions. Second, the variability in study designs, patient populations, and outcome definitions hindered formal meta-analysis. Third, many studies lacked data on critical confounding factors, such as disease severity, comorbidities, and intraoperative variables, which may influence outcomes. Fourth, in some studies, factors such as imaging-confirmed nerve root compression were applied as inclusion criteria to define study populations rather than true surgical indications. As such, our synthesis reflects both explicit indications and de facto criteria used to select patients for participation. Additionally, publication bias could not be definitively excluded. Finally, the review protocol was not prospectively registered on PROSPERO, which should be considered when interpreting the findings.

## 6 Conclusion

This systematic review highlights the significant variability in surgical indications, timing, and clinical decision-making for lumbar disc herniation. While imaging-confirmed nerve root compression and severe or refractory pain were the most consistent indications for surgery, thresholds for motor and sensory deficits varied widely, reflecting a lack of standardization. Similarly, the criteria for transitioning from conservative to surgical treatment demonstrated substantial heterogeneity, with timing thresholds ranging from as early as two weeks for neurological deterioration to over six weeks for persistent symptoms. The Index of Qualitative Variation underscores the fragmented nature of clinical practice, revealing high variability in definitions of conservative treatment failure, motor deficits, and sensory deficits across studies. These inconsistencies are compounded by variability in study methodologies, patient characteristics, and risk of bias, further challenging efforts to establish robust, standardized guidelines for LDH management. In summary, the findings of this review emphasize the urgent need for standardized definitions, particularly for conservative treatment failure and motor deficit severity (IQV = 0.96), and precise guidelines to optimize clinical decision-making for LDH. Establishing consensus across surgical indications and timing criteria will be essential for improving patient outcomes and advancing the quality of care in this critical area of spine surgery. Once standardization is achieved, future research should focus on conducting high-quality randomised controlled trials incorporating emerging technologies to advance the quality of care for LDH patients.

## Funding

No funding was received for this study.

## Competing of interest

The authors declare that they have no known competing financial interests or personal relationships that could have appeared to influence the work reported in this paper.
