# Supplementary material for: Indications for surgery versus conservative treatment in the management of lumbar disc herniations: A systematic review
Source: Brain Spine. 2025 Sep 27;5:105619. doi: 10.1016/j.bas.2025.105619 (PMC12513110; doi:10.1016/j.bas.2025.105619)

**SUPPLEMENTAL DIGITAL CONTENT**

**Indications for Surgery Versus Conservative Treatment**

**In the Management of Lumbar Disc Herniations:**

**Systematic Review**

Santhosh G. Thavarajasingam^1,4,5^ ; Ahmed Salih^1,2^

Aksaan Arif^1,2^; Madhur Varadpande^1,2^; Pratheeshan Sabeshan^1,2^

Hariharan Subbiah Ponniah^1,2^; Sree Kanakala^1,2^; Srikar R. Namireddy^1,2^ ; Daniele S. C. Ramsay^1,2^

Ahkash Thavarajasingam^1,3^; Daniel Scurtu^4^ ; Dragan Jankovic^4^ ; Andreas Kramer^4,5^

Florian Ringel^4,5^

**INSTITUTION:**

1. Imperial Brain & Spine Initiative, Imperial College London, London, United Kingdom

2. Faculty of Medicine, Imperial College London, London, United Kingdom

3. Faculty of Medicine, Medizinische Hochschule Hannover, Hannover, Germany.

4. Department of Neurosurgery, LMU University Hospital Munich, Munich, Germany

5. EANS Spine Section, European Association of Neurosurgical Societies, Europe

Table of Contents

[**Supplementary Table 1:** Search strategy. 3](#_Toc183115483)

[**Supplementary Table 2:** Inclusion and exclusion criteria. 8](#_Toc183115484)

[**Supplementary Table 3:** Table of extracted variables. 9](#_Toc183115485)

[**Supplementary Table 4**: Level of evidence based on the Oxford Centre of Evidence-Based Medicine (OCEBM) Levels of Evidence. 10](#_Toc183115486)

[**Supplementary Figure 1**: Risk of bias analysis of all included studies (NOS). 12](#_Toc183115487)

[**Supplementary Figure 2**: Risk of bias analysis of all included studies (ROB-1 tool). 13](#_Toc183115488)

# **Supplementary Table 1:** Search strategy and short excerpt.

| **Database** | **Full Search Strategy Terms** | **Publication Dates** | **Results (n)** |
| --- | --- | --- | --- |
| Medline | (lumbar disc herniation/ or intervertebral disc displacement/ or (lumbar herniation or lumbar disc disease or herniated disc or slipped disc or disc prolapse or disc protrusion or disc extrusion or disc sequestration or disc rupture or disc derangement or nucleus pulposus herniation or nucleus pulposus prolapse or disc displacement or intervertebral disc herniation or lumbar disc pathology) AND  surgery/ or surgical procedures, operative/ or (surg* or surgery or operative management or surgical intervention or operative intervention or decompression surg* or discectomy or laminectomy or microdiscectomy or spinal fusion or surgical treatment or surgical management or surgical approach or surgical decompression or spine surg* or operative procedures or surgical techniques or operative treatment or spinal surg* or back surg* or spinal decompression or minimally invasive surg* or minimally invasive spinal surg*) AND  (surgical indication or indication for surg* or criteria for surg* or surgical criteria or operative indication or surgical need or need for surg* or surgery requirement or surgical decision or decision for surg* or surgical necessity or necessity for surg* or criteria for operative intervention or operative decision-making or surgical threshold or when to operate or patient selection for surg* or urgent surg* or emergency surg* or acute surg* or neurological deficit or motor deficit or motor weakness or cauda equina syndrome or cauda equina or radiculopathy or bladder dysfunction or bowel dysfunction or muscle weakness or foot drop or paralysis or paresthesia or sensory loss or neurological symptoms or neurological impairment). | 1946 - 2024 | n = 925 |
| Embase | (lumbar disc herniation/ or intervertebral disc displacement/ or (lumbar herniation or lumbar disc disease or herniated disc or slipped disc or disc prolapse or disc protrusion or disc extrusion or disc sequestration or disc rupture or disc derangement or nucleus pulposus herniation or nucleus pulposus prolapse or disc displacement or intervertebral disc herniation or lumbar disc pathology) AND  surgery/ or surgical procedures, operative/ or (surg* or surgery or operative management or surgical intervention or operative intervention or decompression surg* or discectomy or laminectomy or microdiscectomy or spinal fusion or surgical treatment or surgical management or surgical approach or surgical decompression or spine surg* or operative procedures or surgical techniques or operative treatment or spinal surg* or back surg* or spinal decompression or minimally invasive surg* or minimally invasive spinal surg*) AND  (surgical indication or indication for surg* or criteria for surg* or surgical criteria or operative indication or surgical need or need for surg* or surgery requirement or surgical decision or decision for surg* or surgical necessity or necessity for surg* or criteria for operative intervention or operative decision-making or surgical threshold or when to operate or patient selection for surg* or urgent surg* or emergency surg* or acute surg* or neurological deficit or motor deficit or motor weakness or cauda equina syndrome or cauda equina or radiculopathy or bladder dysfunction or bowel dysfunction or muscle weakness or foot drop or paralysis or paresthesia or sensory loss or neurological symptoms or neurological impairment). | 1947 - 2024 | n = 1255 |
| Scopus | ("lumbar disc herniation" OR "intervertebral disc displacement" OR "lumbar herniation" OR "lumbar disc disease" OR "herniated disc" OR "slipped disc" OR "disc prolapse" OR "disc protrusion" OR "disc extrusion" OR "disc sequestration" OR "disc rupture" OR "disc derangement" OR "nucleus pulposus herniation" OR "nucleus pulposus prolapse" OR "disc displacement" OR "intervertebral disc herniation" OR "lumbar disc pathology")  AND  (surg* OR surgery OR "operative management" OR "surgical intervention" OR "operative intervention" OR "decompression surgery" OR discectomy OR laminectomy OR microdiscectomy OR "spinal fusion" OR "surgical treatment" OR "surgical management" OR "surgical approach" OR "surgical decompression" OR "spine surgery" OR "operative procedures" OR "surgical techniques" OR "operative treatment" OR "spinal surgery" OR "back surgery" OR "spinal decompression" OR "minimally invasive surgery" OR "minimally invasive spinal surgery")  AND  ("surgical indication" OR "indication for surg*" OR "criteria for surg*" OR "surgical criteria" OR "operative indication" OR "surgical need" OR "need for surg*" OR "surgery requirement" OR "surgical decision" OR "decision for surg*" OR "surgical necessity" OR "necessity for surg*" OR "criteria for operative intervention" OR "operative decision-making" OR "surgical threshold" OR "when to operate" OR "patient selection for surg*" OR "urgent surg*" OR "emergency surg*" OR "acute surg*" OR "neurological deficit" OR "motor deficit" OR "motor weakness" OR "cauda equina syndrome" OR "cauda equina" OR radiculopathy OR "bladder dysfunction" OR "bowel dysfunction" OR "muscle weakness" OR "foot drop" OR paralysis OR paresthesia OR "sensory loss" OR "neurological symptoms" OR "neurological impairment") | 1943 - 2024 | n = 2410 |
| Pubmed | (lumbar disc herniation/ or intervertebral disc displacement/ or (lumbar herniation or lumbar disc disease or herniated disc or slipped disc or disc prolapse or disc protrusion or disc extrusion or disc sequestration or disc rupture or disc derangement or nucleus pulposus herniation or nucleus pulposus prolapse or disc displacement or intervertebral disc herniation or lumbar disc pathology) AND  surgery/ or surgical procedures, operative/ or (surg* or surgery or operative management or surgical intervention or operative intervention or decompression surg* or discectomy or laminectomy or microdiscectomy or spinal fusion or surgical treatment or surgical management or surgical approach or surgical decompression or spine surg* or operative procedures or surgical techniques or operative treatment or spinal surg* or back surg* or spinal decompression or minimally invasive surg* or minimally invasive spinal surg*) AND  (surgical indication or indication for surg* or criteria for surg* or surgical criteria or operative indication or surgical need or need for surg* or surgery requirement or surgical decision or decision for surg* or surgical necessity or necessity for surg* or criteria for operative intervention or operative decision-making or surgical threshold or when to operate or patient selection for surg* or urgent surg* or emergency surg* or acute surg* or neurological deficit or motor deficit or motor weakness or cauda equina syndrome or cauda equina or radiculopathy or bladder dysfunction or bowel dysfunction or muscle weakness or foot drop or paralysis or paresthesia or sensory loss or neurological symptoms or neurological impairment). | 1943 - 2024 | n = 1686 |

In Supplementary Table 1 the search strategy performed on 10^th^ August 2024 is shown below outlining the respective databases, the search terms, publication dates chosen as limiting factors, and number of results from each database are shown.

# **Supplementary Table 2:** Inclusion and exclusion criteria.

| **Inclusion criteria** | **Exclusion criteria** |
| --- | --- |
| - Published in the English language - Peer-reviewed journals - Adult human patients with lumbar disc herniation (see search string for synonyms) - Studies evaluating indications (including timing of surgery, motor deficits, failure of conservative therapy) for surgical treatment | - All non-English languages - Commentaries, case reports, narrative reviews, letters to editors, books - Any animal studies and lab-based studies - Studies on children and adolescents (<18 years) - Patient with cauda equina syndrome |

In Supplementary Table 2, the inclusion and exclusion criteria used when filtering studies based off search results are shown.

# **Supplementary Table 3:** Table of extracted variables.

| **Extracted variables** |
| --- |
| - Study Characteristics: Title, DOI, country, publication year, first author, study design, and sample size. - Treatment Details: Type of conservative therapy, duration of conservative therapy, surgical approach, and indications for surgery. - Indications of surgical intervention - Outcome Measures: Conservative treatment success rates, pain reduction (using the Visual Analog Scale [VAS]), and surgical success rates. - Adverse Effects: Complications and adverse effects reported for both surgical and conservative treatments. |

In Supplementary Table 3, a table shows the extracted variables.

# **Supplementary Table 4**: Level of evidence based on the Oxford Centre of Evidence-Based Medicine (OCEBM) Levels of Evidence.

| **Study** | **OCEBM Level** |
| --- | --- |
| Gurung et al. 2023 | 2b |
| Motiei-Langroudi et al. 2023 | 3b |
| Bailey et al. 2021 | 1b |
| Thomé et al. 2022 | 2b |
| Gupta et al. 2020 | 3b |
| Ostafinski et al. 2020 | 3b |
| Petr et al. 2019 | 2b |
| Kim et al. 2016 | 3b |
| Motiei-Langroudi et al. 2014 | 3b |
| Overdevest et al. 2014 | 1b |
| Choi et al. 2013 | 2b |
| Haugen et al. 2012 | 3b |
| Lurie et al. 2013 | 2b |
| Thomas et al. 2007 | 2b |
| Carlisle et al. 2005 | 3b |
| Rothoerl et al. 2002 | 2b |
| Kavuncu et al. 2001 | 2b |
| Weinstein et al. 2006 | 1b |
| Weinstein et al. 2006 | 2b |
| Peul et al., 2007 | 1b |

Supplementary Table 4 shows the results of the Oxford Centre of Evidence-Based Medicine (OCEBM) Levels of Evidence tool.

# **
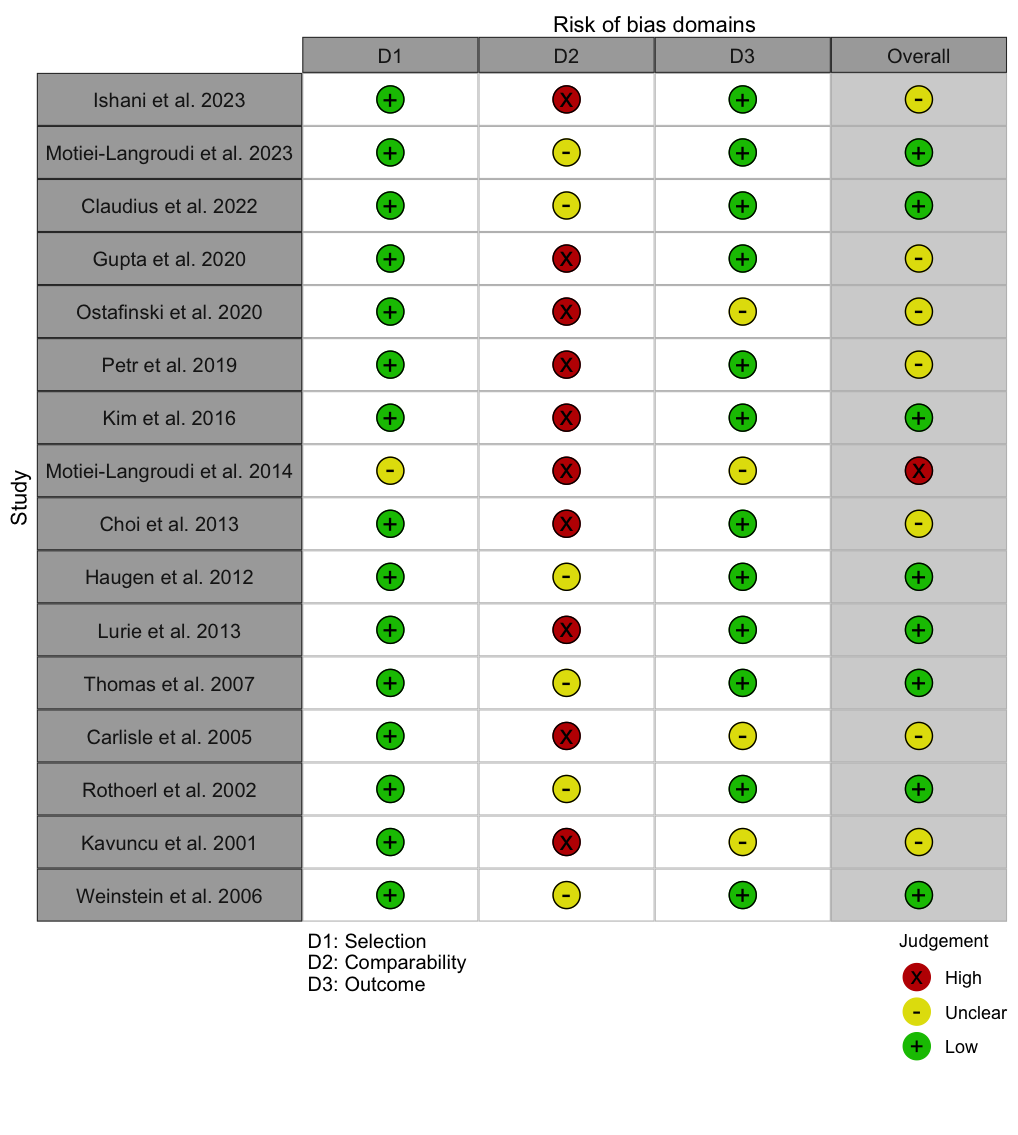
Supplementary Figure 1**: Risk of bias analysis of all included non-randomized studies using the Newcastle-Ottawa Scale (n=16).

# **Supplementary Figure 2**: Risk of bias analysis of all included randomized studies using the ROB-1 tool (n=4).


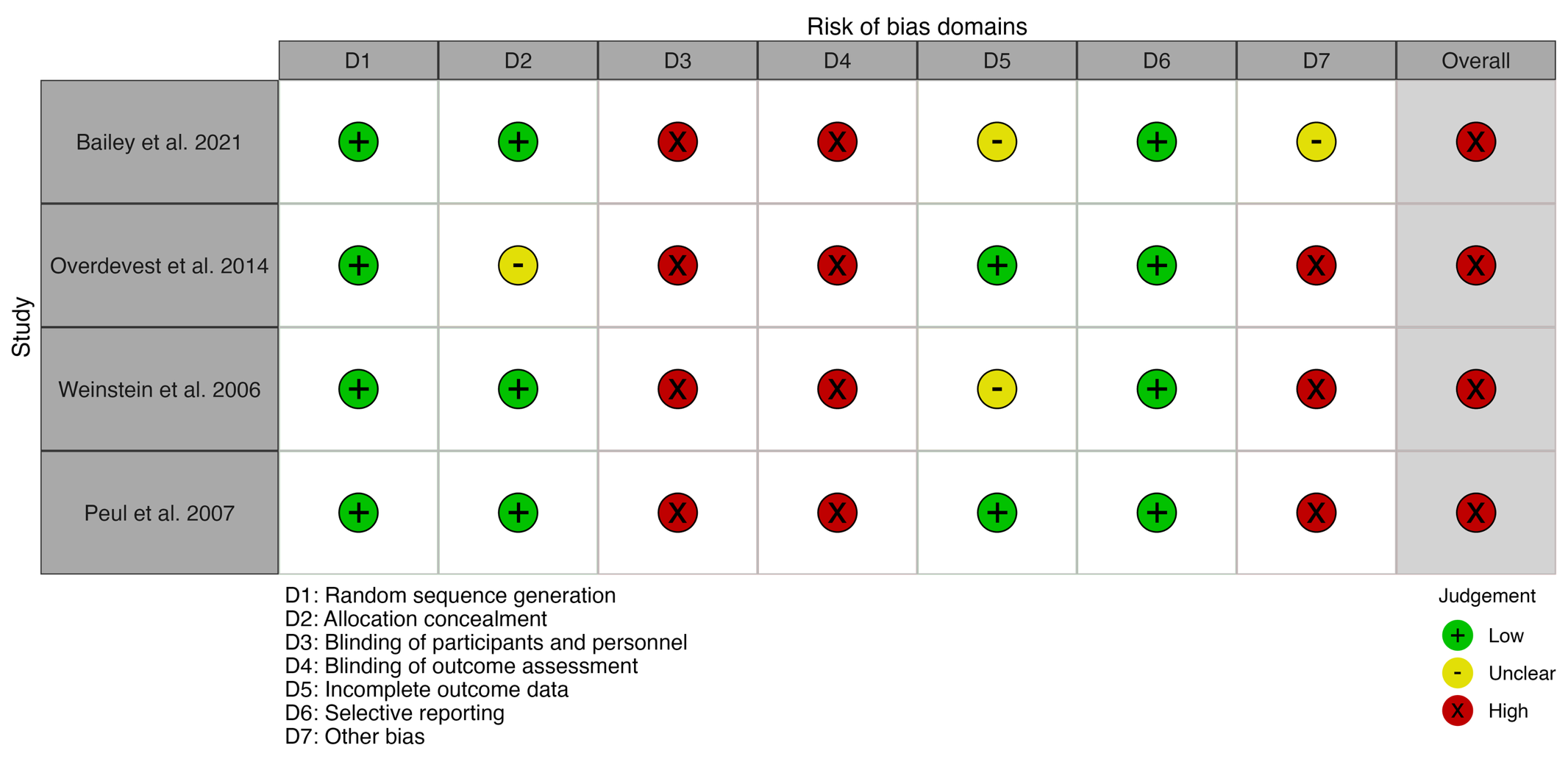

Supplement: Multimedia component 1 [file mmc1.docx]
